# Autonomous Dynamic Control of Crown Ether Cargo Release from [2]Rotaxane Carriers in a Piperidine Oscillator

**DOI:** 10.1021/jacs.5c05460

**Published:** 2025-06-18

**Authors:** Kamil D. Petryczkiewicz, Johanan Kootstra, Maëlle Le Cacheux, Aleksei Tsygankov, Jan L. Sneep, Matthijs ter Harmsel, Syuzanna R. Harutyunyan

**Affiliations:** Stratingh Institute for Chemistry, 3647 University of Groningen, Groningen 9747 AG, The Netherlands

## Abstract

Chemical oscillations play a fundamental role in biological systems, yet their synthetic counterparts remain challenging to implement with functional outputs. Here, we report a piperidine-based chemical oscillator that autonomously drives periodic cleavage of a [2]­rotaxane carrier, leading to controlled cargo release. The system operates through self-sustained oscillations, triggering rotaxane cleavage and the release of a crown ether cargo. Crown ethers were selected for their reactivity distinct from piperidine, while Fmoc-protected benzylamine rotaxane structures allowed for straightforward carrier modification. For all tested carriers, a piperidine pulse is present and occurs simultaneously with carrier cleavage, yielding up to 95% cargo release. Under flow conditions, periodic cargo release was sustained without extensive reoptimization, demonstrating the robustness of the system. Additionally, by adjusting space velocity, trigger concentration, and inhibitor levels, the oscillation period was varied by up to 2.5 h, with cargo release amplitude changing more than 3-fold. This work demonstrates the potential of catalytic oscillators to regulate downstream processes, paving the way toward construction of complex dynamic chemical systems.

## Graphical abstract

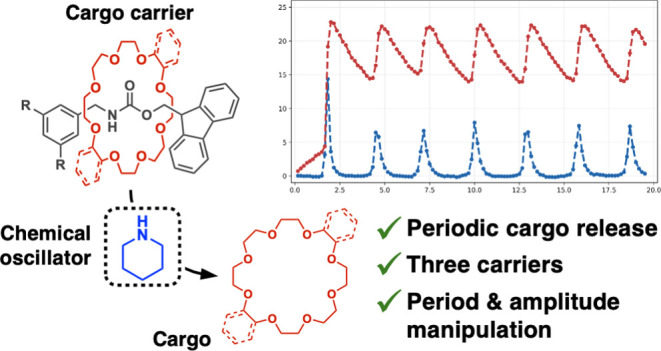

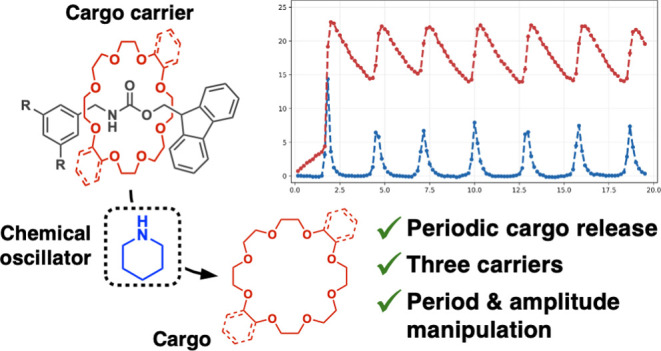

## Introduction

Biological systems exhibit remarkable temporal control, with a variety of naturally occurring processes characterized by periodic changes in time across wide ranges of duration and size. Examples such as the lunar cycle, circadian rhythms, cardiac rhythms, and metabolic pathways all underscore the importance of oscillatory phenomena in nature.^1–5^ These oscillations are intricately integrated into hierarchical systems, where they interact with functional carriers to regulate complex biological processes (Figure 1a).^6^ Intracellular calcium oscillations exemplify this principle, as the periodic release of calcium ions functions as a signal for other processes to commence.^7^ These oscillations are transmitted by calcium-binding proteins, such as calmodulin, to downstream effectors like kinases and phosphatases, regulating essential processes including muscle contraction, neurotransmitter release, and gene transcription.^8^ Similarly, metabolic oscillations, mediated by NADH, ATP and their associated enzymes, affect critical pathways such as glycolysis and oxidative phosphorylation.^9^ Moreover, pulsatile release of hormones, including calcium-driven insulin secretion by pancreatic β-cells, reveals the role of oscillatory control in the strict regulation of blood glucose levels.^10^

**Figure 1:**
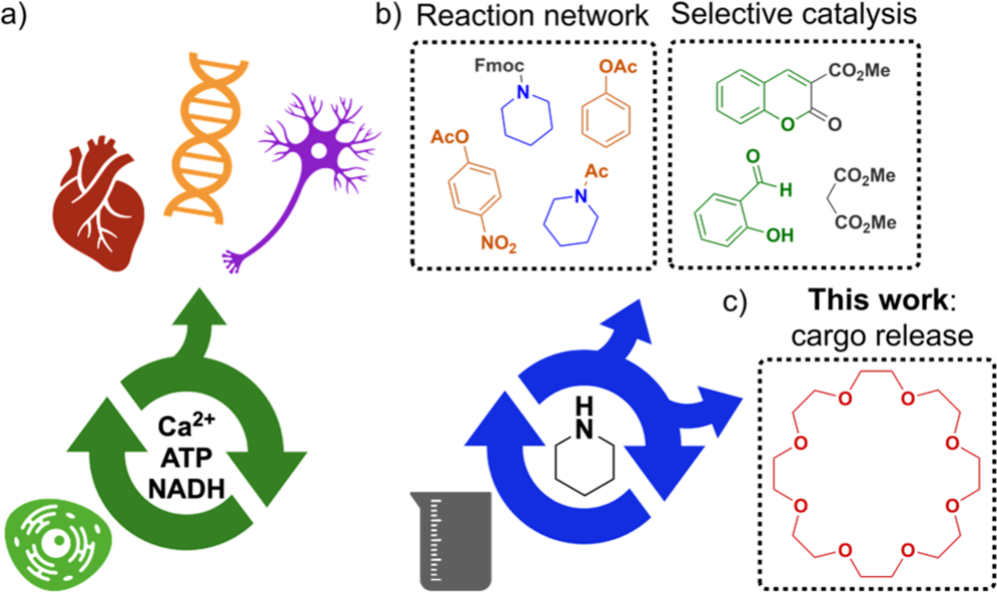
(a) Role of natural oscillations observed in biological systems. (b) Overview of the synthetic piperidine oscillator. (c) Autonomous release of cargo by the piperidine oscillator.

These and other naturally occurring oscillations have inspired the design of synthetic systems that seek to emulate the temporal control observed in biological processes for potential applications in medicine, biotechnology and material science.^11^

For instance, regenerative medicine could benefit from oscillatory growth factor release, mimicking natural signaling to guide tissue repair and cell differentiation.^11a^ Likewise, pulsatile drug delivery, inspired by hormonal rhythms, promises improved therapeutic outcomes for conditions benefiting from chronopharmacotherapy, such as cancer, diabetes or cardiovascular disorders.^12^ Finally, synthetic oscillators can also serve as fundamental models of more complex biological systems.^13^

Despite their promise, chemically coupling the kinetics of oscillators to downstream reaction pathways within a single system remains underexplored. Enabling functions such as triggering another chemical reaction or facilitating the periodic release of a cargo or signaling molecule is a challenge compounded by two major limitations. First, synthetic oscillators must maintain their intrinsic timekeeping function while participating in new roles - a balance often disrupted in stoichiometric reactions where oscillator components are consumed.^14^

Second, achieving biocompatibility, structural modularity, and functional versatility remains a significant hurdle. Most synthetic oscillators depend on redox chemistry and require harsh conditions, such as extreme pH or highly oxidative environments, which significantly narrows their potential application window.^15^ Emerging alternatives, such as oscillatory networks based on biomolecules, supramolecular assemblies, and organic molecules, offer promising avenues but require further advancement.^16^

In this context, our group has recently developed the first chemical oscillator based on the small organic molecule piperidine: a catalyst (Figure 1b).^17^ Embedding a catalytic functionality within the oscillator was essential to couple its kinetics to an additional reaction, while preserving its time-keeping ability, as the transient nature of catalysis imposes minimal load on the system. The resulting oscillator is capable of autonomously and periodically catalyzing the independent Knoevenagel condensation reaction.

Building on the modular design, structural simplicity, and catalytic capabilities of the piperidine-based oscillator, we sought to explore the possibility of exploiting oscillatory kinetics to drive a cargo release mechanism, in analogy to biological mechanisms for signaling molecule release. Extensive research has led to several strategies for cargo release, where diverse cargo and carrier designs have been investigated.^18^ However, most of these systems are designed for single-event cargo release and are thus inherently nonautonomous and lack periodicity, except for a palladium-functionalized macrogel system, where pH oscillations trigger the periodic release of fluorescein.^19^ Synchronizing the repeated release of a functional cargo with the kinetics of an oscillating system could establish a framework that emulates the dynamic, coupled processes inherent to biological systems.

Motivated by this idea, we demonstrate that our piperidine-based oscillator can cleave a [2]­rotaxane carrier molecule, leading to the autonomous periodic release of a crown ether cargo (Figure 1c). This opens the door to coupling oscillatory kinetics with functions intrinsic to crown ethers and distinct from the reactivity of piperidine.

## Results and Discussion

### Design of the Cargo Release System

To function, our previously reported catalytic oscillator utilizes Fmoc-protected piperidine (Fmoc-pip) as fuel, which is the source of free piperidine within the system. The release of piperidine is achieved in two ways: a catalytic reaction with *N*-methylpiperidine (trigger) and the autocatalytic deprotection by piperidine itself. To regulate the release, two types of inhibitors are employed in the form of acetylating agents: 4-nitrophenyl acetate (fast inhibitor) and phenyl acetate (slow inhibitor). With this network architecture (Figure 2a), each oscillatory cycle can be described by three phases: a lag phase, autocatalysis and decay (Figure 2b). The autocatalytic production of piperidine from Fmoc-pip is initiated by a slow trigger reaction. This initial piperidine is rapidly consumed by fast inhibition (via *p*-nitrophenyl acetate), which proceeds more quickly than the autocatalytic process. This introduces a delay, preventing premature autocatalysis and allowing piperidine to accumulate only after the fast inhibitor is depleted. Meanwhile, a slow inhibition reaction (via phenyl acetate) is also active from the start but proceeds at a much lower rate than both fast inhibition and autocatalysis. As a result, its effect becomes apparent only after the autocatalytic burst, once Fmoc-pip is exhausted. This results in gradual reduction in piperidine concentration, returning the system to baseline. Importantly, the trigger is orthogonal to both inhibitors, therefore preserving its initiating role throughout. In a batch (closed) system, this interplay results in a single piperidine concentration pulse. Under continuous flow conditions (CSTR), where fuel, trigger and inhibitors are constantly replenished, the system can be maintained out of equilibrium, enabling repeated oscillations, but only within an appropriate window of reaction parameters.

**Figure 2:**
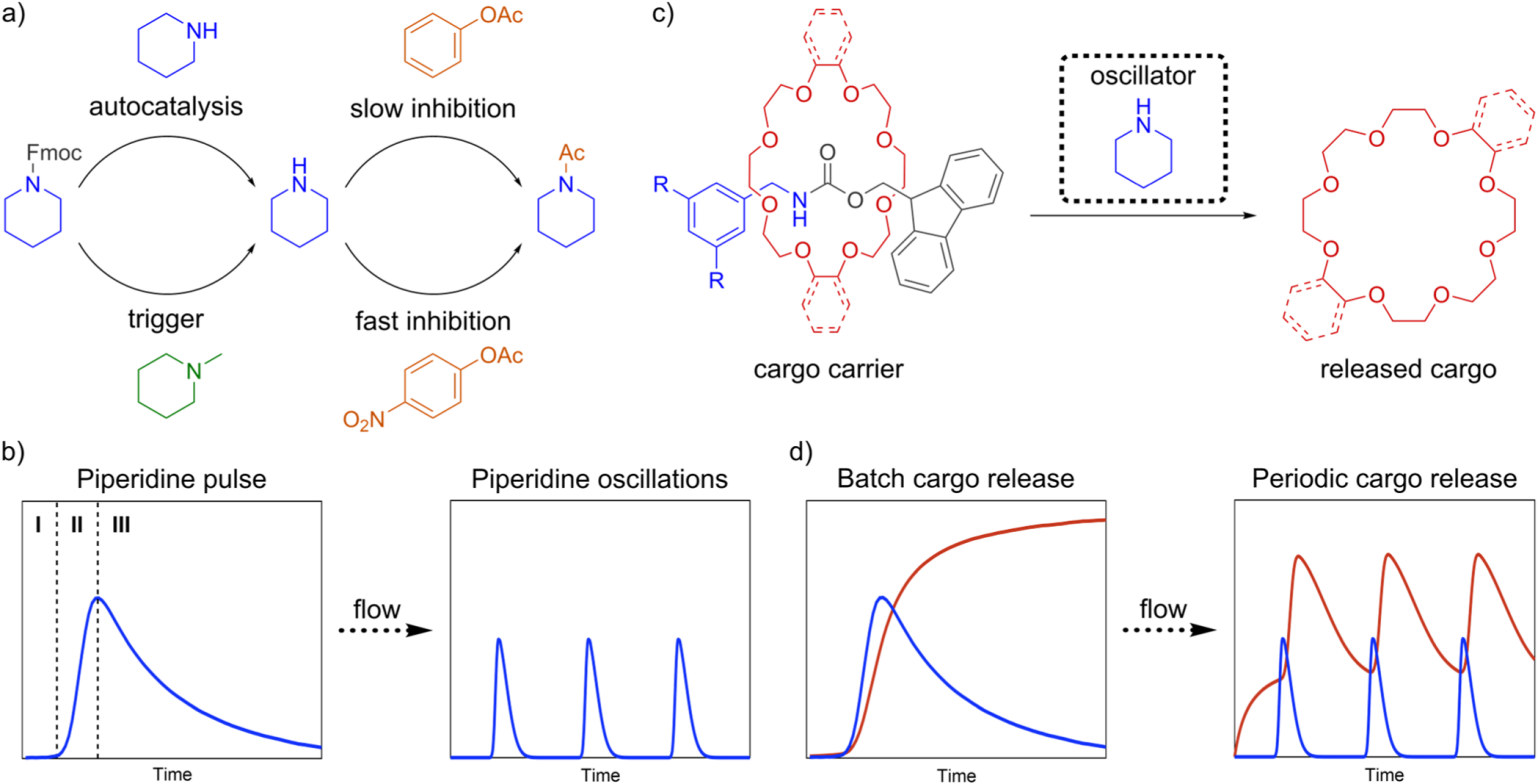
(a) Piperidine oscillator components and their interactions. (b) Variation of piperidine concentration over time when the system is initiated in batch and flow conditions. The piperidine pulse obtained in batch conditions can be split into three phases: I–lag phase, II–autocatalytic phase, and III–decay phase. (c) Outline of the designed cargo release system, where the Fmoc-protected benzylamine [2]­rotaxane carrier is cleaved by piperidine, resulting in the release of a crown ether cargo molecule. (d) Variation of piperidine (blue trace) and cargo (red trace) concentration over time when the cargo release system is initiated under batch and flow conditions.

To achieve autonomous periodic release of a functional cargo driven by oscillatory kinetics, we envisioned a reaction network in which a cargo-bearing carrier introduced to the oscillator is cleaved by piperidine (Figure 2c). Because the concentration of piperidine varies periodically over time, the cargo would be released in synchronicity with the peaks in the piperidine concentration (Figure 2d). This way one mode of action, i.e. piperidine base catalysis, can induce the emergence of a new reactivity type, which comes from the properties of the released cargo. However, to realize such a system, both the carrier and the cargo need to fulfill several conditions: *(*a*) The carrier must undergo catalytic cleavage by piperidine; (b) Neither the carrier nor the waste products substantially interfere with the core oscillator; (c) The cargo must display a mode of action orthogonal to that of piperidine base catalysis to extend the oscillator’s functionality.*

In search of suitable candidates, we took inspiration from supramolecular chemistry and extensive research of interlocked molecules such as rotaxanes. Their simplest design includes an axle with a macrocycle trapped around it, enabling a wide range of dynamic properties^18a,20^ In these systems, crown ethers are a popular choice for the macrocycle motif, while they also display a variety of applications ranging from phase transfer and nucleophilic catalysis to ion recognition.^21^ These properties allow to introduce a new reactivity mode into the system, beyond base or nucleophilic catalysis. Furthermore, a recent discovery by the Leigh group showed that crown ethers can also promote the formation of rotaxanes via a metal-free active templating effect.^22^ One of the rotaxanes obtained this way was Fmoc-protected 3,5-bis­(trifluoromethyl)­benzylamine (TFBA) with 24-crown-8 ether (24C8) as the macrocycle.^22b^ This drew our attention as the Fmoc scaffold is an important motif in fuel molecules for molecular machines, as well as for our piperidine oscillator.^17,23^ Furthermore, the fuel itself is kinetically stable, which enables control over the decomposition process using a base such as piperidine.^23^

With these considerations in mind, we decided to utilize crown ether-bearing Fmoc-based rotaxanes as carriers for the cargo release system (Figure 2c). This design not only allows the crown ether cargo release by catalytic deprotection with piperidine but also releases waste products (CO_2_ and DBF) already present in the reaction mixture.

To build the cargo release system, we chose Fmoc-TFBA 24C8 [2]­rotaxane (Figure 3, carrier **1**) as the model carrier molecule. However, to provide a broader scope of carriers and test the robustness of the oscillator, we also wanted to probe other rotaxanes with an Fmoc motif. For this purpose, we decided to introduce modifications on the amine and crown ether parts of the carrier molecules. The amine-end modification is possible, provided the amine is primary and bulky enough to prevent macrocycle dethreading. Since the nucleophilicity of the used amine is expected to influence the degree of interference with the core oscillator, we chose 3,5-di-*tert*-butylbenzylamine (TBBA), which is more nucleophilic than TFBA, as an alternative amine-end (carrier **2**). Finally, we decided to introduce the bulkier dibenzo-24-crown-8 ether (db24C8) as a modified macrocycle (carrier **3**), where the benzene rings can serve as handles for future modification.^24^ With the design of the cargo release system and the cargo and carrier architecture in hand, we proceeded with synthesis of carrier molecules.

**Figure 3:**
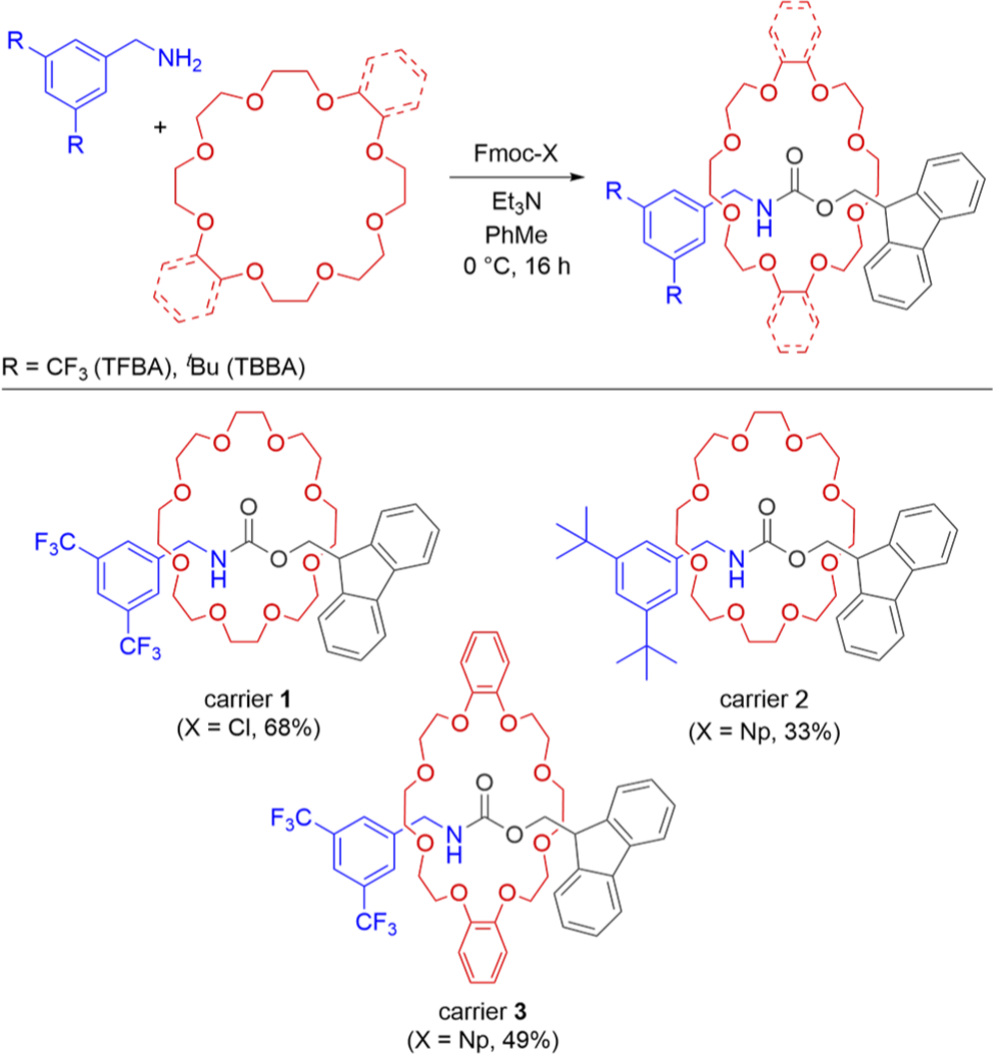
Synthesis and structure of three carrier molecules. Reaction conditions: in toluene 0.22 M benzylamine, crown ether (1 equiv), Fmoc-X (1 equiv), Et_3_N (2 equiv) at 0 °C for 16 h.

### Synthesis

Our starting point was the active template synthesis of **1** reported in the literature.^22b^ However, the same protocol did not allow for efficient synthesis of carriers **2** and **3**. This was solved by changing the electrophile to the Fmoc 4-nitrophenyl ester (Fmoc-Np), which resulted in yields comparable to the original procedure (Supporting Information Table S1).

### Batch Experiments

Having successfully synthesized all three carriers, we investigated their effect on the piperidine system under batch conditions (Figure 4a).

**Figure 4:**
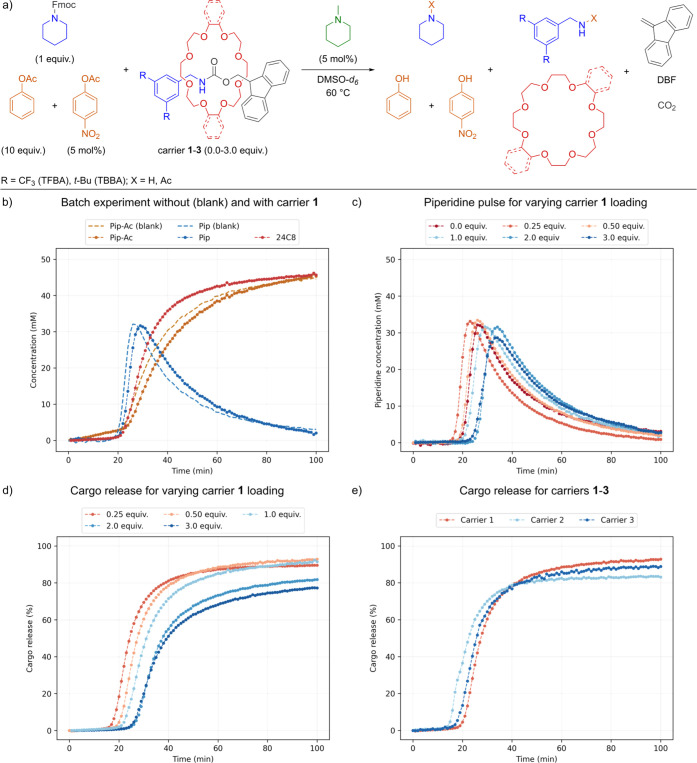
Reaction scheme and results of cargo release experiments under batch conditions. Data points are reported as averages of two runs. Reaction conditions: 50 mM Fmoc-pip, carriers **1**–**3** (0.0–3.0 equiv), trigger (5 mol %), fast inhibitor (5 mol %), slow inhibitor (10 equiv) in DMSO-*d*_*6*_ at 60 °C for 100 min. Monitored by ^1^H NMR spectroscopy with an internal standard from the moment of trigger addition (*t* = 0 min). (a) Reaction network initiated under batch conditions with carrier **1**. (b) Concentration of piperidine (blue), 24C8 (red), and piperidine acetate (Pip-Ac, yellow) over time in a batch experiment with no carrier (blank) and 1.0 equiv of carrier **1**. (c) Piperidine concentration over time for different carrier **1** loadings (0.0–3.0 equiv). (d) Cargo release over time for different carrier **1** loadings (0.0–3.0 equiv). (e) Cargo release over time for carriers **1**–**3** (0.5 equiv).

As an initial trial, we used Fmoc-pip (50 mM), carrier **1** (1.0 equiv) and fast and slow inhibitors (5 mol % and 10 equiv respectively) in DMSO-*d*_*6*_ at 60 °C. The reaction was initiated by the addition of trigger (5 mol %) and the concentrations were monitored via ^1^H NMR spectroscopy. Importantly, a piperidine pulse (Figure 4b, blue trace) exhibiting the three characteristic phases-lag phase, autocatalysis, and decay - was observed. The lag phase was approximately 3 min longer compared to the “blank” piperidine pulse, i.e. in the absence of the carrier molecule, indicating that the reaction was only slightly influenced by the carrier. Moreover, we were excited to observe that after 100 min, over 90% of the crown ether cargo had been successfully released from the carrier, with the release initiating precisely at the onset of the piperidine concentration increase (Figure 4b, red trace vs blue).

To better understand interactions within this system, we conducted a series of control experiments. First, we investigated the reactivity of TFBA with the inhibitors used for the oscillator and established that TFBA reacts rapidly with the fast inhibitor, but much more slowly with the slow inhibitor compared to piperidine (Supporting Information Figures S5 and S7), indicating that TFBA could only influence the lag phase.

To isolate the impact of the crown ether, we performed a piperidine pulse experiment without the addition of carrier but with 24C8 (20 mol %), which resulted in an unchanged reaction profile, suggesting that the crown ether itself does not interfere with the process (Supporting Information Figure S9). Finally, we monitored the reaction of TFBA with the slow inhibitor in the presence of 24C8 and observed no change in the acylation rate (Supporting Information Table S4), despite literature reports showing increased amine nucleophilicity in the presence of crown ethers in toluene.^22c^ We hypothesize that this discrepancy arises because our experiments were conducted in DMSO, where strong solvent-amine interactions likely make crown ether coordination to the amine less favorable. Additionally, crown ethers can form complexes with DMSO itself, further diminishing interactions with amines.^25^

Next, to assess how much carrier loading the oscillating system can tolerate without significant disruption, we systematically increased the amount of carrier **1** (from 0 to 3 equiv relative to Fmoc-pip) and measured the peak piperidine concentration (amplitude) and the percentage of released cargo after 100 min (Figure 4c,d, Supporting Information Table S2). At low carrier loadings (0.25 equiv), we observed a slightly shorter lag phase and higher piperidine amplitude than in the “blank” piperidine pulse, along with 90% cargo release. However, as the carrier loading increased, the lag phase increased, while both the piperidine amplitude and cargo release gradually decreased. Most likely such a decrease in cargo release is due to an increased amount of carrier being decomposed by a fixed quantity of piperidine within a limited time frame. Nevertheless, even at the highest tested loading (3 equiv), the system still achieved around 77% cargo release, demonstrating its robustness even under relatively high carrier concentrations. For subsequent investigations, we selected 0.5 equiv of carrier, as this condition provided the highest cargo release while maintaining a piperidine pulse most comparable to the “blank” piperidine pulse.

Having characterized the batch cargo release system for carrier **1**, we tested carriers **2** and **3** (0.5 equiv) and determined changes in the pulse profiles (Figure 4e, Supporting Information Table S3). Results revealed that the cargo release was lowered from 93% for carrier **1** to 83% for carrier **2**, indicating that the latter is deprotected more slowly, possibly due to steric effects. Moreover, although the piperidine amplitude remained unchanged, the lag phase duration decreased by 6 min. This is most likely due to the reactions of TBBA with the inhibitors, fuel, and carrier itself. Thus, to control such a complex network, it is essential to use weakly reactive amines and suppress the additional auto- and cross-catalytic processes. On the other hand, for carrier **3** we observed only minor changes of both the lag phase and piperidine amplitude, demonstrating that both 24C8 and db24C8 are compatible with the oscillator and the increased bulk only moderately influences the system.

**Figure 5:**
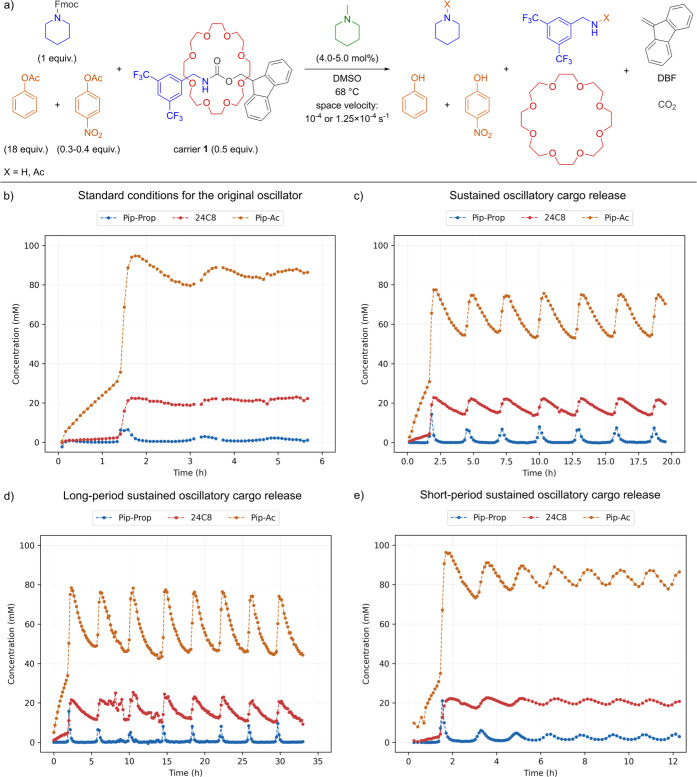
Reaction scheme and the results of cargo release in flow experiments. Reaction conditions: 100 mM Fmoc-pip, carrier **1** (0.50 equiv), trigger (4.0–5.0 mol %), fast inhibitor (0.30–0.40 equiv), slow inhibitor (18 equiv) in DMSO at 68 °C with space velocity: *v* = 10^–4^ s^–1^ or 1.25 × 10^–4^ s^–1^. Monitored by sampling, quenching with 4-nitrophenyl propionate, and analysis via GC-FID with an internal standard. (a) Reaction network initiated under flow conditions with carriers **1**. (b) Damped oscillations with carrier **1** for: *v* = 10^–4^ s^–1^, trigger (5.0 mol %), and fast inhibitor (0.30 equiv). (c) Sustained oscillations with carrier **1** for: *v* = 10^–4^ s^–1^, trigger (4.5 mol %), and fast inhibitor (0.35 equiv). Only the first 19 h are shown, as then the system switched to a new limit cycle (Supporting Information Figure S13). (d) Sustained oscillations with carrier **1** for: *v* = 10^–4^ s^–1^, trigger (4.5 mol %), and fast inhibitor (0.40 equiv). (e) Sustained oscillations with carrier 1 for *v* = 1.25 × 10^–4^ s^–1^, trigger (4.5 mol %), and fast inhibitor (0.30 equiv).

### In-Flow Experiments

Transforming a single pulse into a series of oscillations requires out-of-equilibrium conditions. This is achieved by placing the system in a continuous stirred tank reactor (CSTR), where fresh starting materials are continuously supplied, and the reaction mixture is continuously removed, maintaining a dynamic steady state. However, sustained oscillations only occur for a finely tuned set of starting conditions, which include initial concentrations and flow rates. In our previous work, we developed a descriptive model to identify conditions that should result in regular and sustained oscillatory behavior.^17^ Since our initial pulse experiments showed minimal interference from the added carrier molecule, we anticipated that the previously established oscillatory conditions could be applied to the current system incorporating the carrier. To test this, we conducted CSTR experiments (Figure 5a) with a space velocity of *v* = 10^–4^ s^–1^ and the following inflow concentrations: Fmoc-pip (100 mM), slow inhibitor (18 equiv), fast inhibitor (0.3 equiv), and trigger (5 mol %), as these conditions were previously shown to support sustained oscillations, and an additional 0.5 equiv of carrier **1**. To prevent the reaction from occurring in the inflow lines, the trigger and the rest of the reactants were continuously supplied to the CSTR through separate feed lines. In our previous study, we reported the reaction temperature of 60 °C as used for batch conditions, however, now we found that due to a temperature probe malfunction, the true temperature was 68 °C, which is why here we also decided to run the reaction at the latter, higher temperature. In the earlier CSTR study, we also monitored concentration changes using in situ infrared spectroscopy, which did not allow for tracking piperidine. To address this limitation, we developed an improved analytical approach based on sample collection and GC-FID analysis.^26^ During collection, samples were diluted and quenched with 4-nitrophenyl propionate, allowing reliable quantification of all key species, including piperidine, which forms piperidine propionate (Pip-Prop). This represents a significant improvement over our previous measurement method, as it enables direct monitoring of piperidine concentrations.

**Figure 6:**
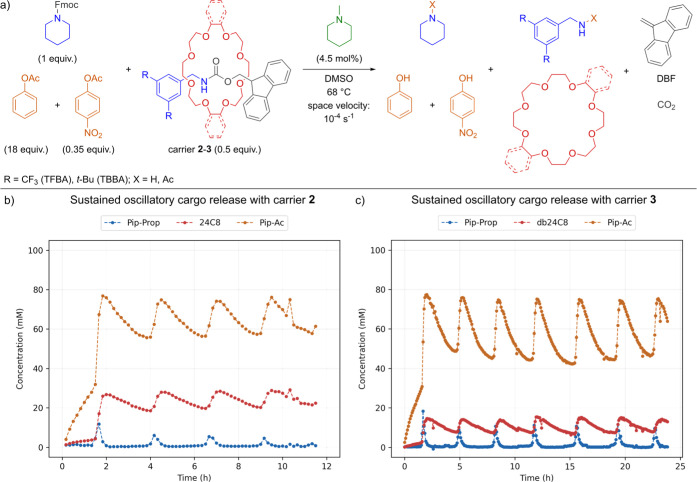
Reaction scheme and the results of cargo release in flow experiments for carriers **2** and **3**. Reaction conditions: 100 mM Fmoc-pip, carriers **2**–**3** (0.50 equiv), trigger (4.5 mol %), fast inhibitor (0.35 equiv), slow inhibitor (18 equiv) in DMSO at 68 °C with space velocity: *v* = 10^–4^ s^–1^. Monitored by sampling, quenching with 4-nitrophenyl propionate, and analysis via GC-FID with an internal standard. (a) Reaction network initiated under flow conditions with carriers **2**–**3**. (b) Sustained oscillations with carrier **2**. (c) Sustained oscillations with carrier **3**.

Unfortunately, oscillations obtained based on the previously found conditions were damped (Figure 5b), indicating that the addition of carrier **1** altered the system. The most likely explanation is that the released TFBA reacted with the continuously added fast inhibitor, reducing piperidine inhibition and disrupting the oscillatory behavior.

To compensate for this effect, we increased the amount of fast inhibitor to 35 mM and lowered the trigger concentration to 4.5 mM. We were delighted to see that this resulted in sustained oscillations and periodic cargo release, although the system switched to a new limit cycle after 19 h (Figure 5c, Supporting Information Figure S13). The latter is suspected to be due to temperature variation and setup fatigue, so for subsequent analysis we focused solely on the first 19 h. We determined that the period was on average 2.8 h and that the mean change of cargo concentration in a cycle (amplitude) was 8.1 mM. Additionally, the mean cargo release was 45%, which is notably lower than in batch experiments due to continuous addition of the fast inhibitor and removal of piperidine. Nevertheless, with only minor changes to the previous conditions we coupled a cargo release process to the oscillator, demonstrating the stability and modularity of the piperidine oscillator.

Encouraged by these results, we aimed to tune the oscillation characteristics (period and amplitude), as such control is crucial for synchronization with downstream processes and potential applications. In general, the oscillation period is primarily determined by the fast inhibitor and trigger concentrations, though they must remain within a certain range to sustain oscillations.^17^ To explore this, we increased the fast inhibitor concentration to 40 mM, which extended the period to 4 h and raised the cargo amplitude to 11 mM (Figure 5d). Conversely, decreasing the fast inhibitor to 30 mM led to damped oscillations (Supporting Information Figure S14). However, the 1.7 h period of these decaying oscillations suggests proximity to viable conditions. To further promote shorter-period oscillations, we increased the space velocity to 1.25 × 10^–4^ s^–1^, which successfully yielded sustained oscillations with a 1.5 h period (Figure 5e).

Notably, cargo release remained high at 44%, only slightly lower than for longer periods, albeit at decreased cargo amplitude (3.3 mM). To counteract this, we lowered the trigger concentration to 4.0 mM, but this resulted in damped oscillations without affecting the period (Supporting Information Figure S15). Finally, we tested carriers 2 and 3 using conditions established for carrier 1 and obtained sustained oscillations for both carriers (Figure 6a–c) without the need of further optimization. In line with the batch experiments, the period and amplitude did not vary significantly between carriers (Supporting Information Table S6). However, we observed noticeable changes in cargo release - increase for carrier 2 to 56% and decrease to 29% for carrier 3 compared to 45% for carrier 1. We have empirically established qualitative trends linking component concentration and space velocity with oscillation period and amplitude. Follow-up quantitative investigations of these effects will make use of *in silico* modeling of the system. Furthermore, improving our experimental setup for better reproducibility is planned as well, and in this regard temperature control and sufficient stirring seem to be key components. Additionally, due to the modularity of the oscillator and carrier, we believe that our system represents a versatile platform for future buildup of complexity. For instance, taking inspiration from our previous work, the crown ether cargo could periodically catalyze a secondary reaction by activating DMSO or a nucleophile by counterion coordination.^21e,25^ However, this design also allows us to consider the system in broader terms, beyond synthetic organic chemistry. For example, utilizing molecular pumps, a network of carriers and loaders could transport the cargo between different axles.^20c,27^ Hence, such a system would allow for artificial, directed small molecule transport, a phenomenon crucial for the functioning of cells.^28^ Another approach would be to use the cargo for periodic (dis)­assembly of supramolecular polymers. The crown ether could noncovalently bind to cationic parts of a polymer and destabilize it, triggering the disassembly.^29^ An interesting concept in this context is the potential to control DNA transcription by periodically changing its melting temperature. This could be achieved by complexing cations that stabilize the negatively charged DNA phosphate backbone or modifying the crown ether with intercalating moieties in likeness to db24C8.^30^ Thus, during periods of low crown ether concentration, the system would be “asleep” (no transcription), while after cargo release the system would become active and begin growth (transcription), making the cargo an artificial transcription factor.^31^

## Conclusions

By rational design of the [2]­rotaxane carrier and exploiting the modularity of the piperidine oscillator, we developed a unique system for the autonomous, periodic release of molecular cargo. The structural similarity of the carrier to the oscillator fuel, combined with the orthogonal crown ether reactivity, allowed the system to function without extensive optimization. Thus, a piperidine pulse in batch conditions could be obtained even when 3 equiv of the carrier **1** were used. Then, after putting the system under flow conditions we achieved sustained oscillations and periodic cargo release from carrier **1**. Importantly, the system can function in several modes, where the cargo release frequency and amplitude are regulated by manipulation of the trigger and inhibitor concentrations. Moreover, increased space velocity promotes high-frequency oscillations, allowing periods as short as 1.5 h. On the other hand, increasing the concentration of the fast inhibitor yielded periods of up to 4 h. What is more, we introduced carriers **2** and **3** into the system without additional condition optimization, resulting in successful batch experiments as well as sustained oscillations with highly similar characteristics to carrier **1**. This constitutes a starting point for future modification of the cargo and using it as a linker with other active molecules. Finally, we hope that this work stimulates future developments in temporal reaction control, periodic catalysis, supramolecular chemistry, as well as biomimetics.

## Supplementary Material



## Notes

Experimental details, materials, and methods, characterization data, additional figures as mentioned in the text, and NMR spectra (PDF)
