# Supplementary material for: Autonomous Dynamic Control of Crown Ether Cargo Release from [2]Rotaxane Carriers in a Piperidine Oscillator
Source: J Am Chem Soc. 2025 Jun 18;147(26):22883–91. doi: 10.1021/jacs.5c05460 (PMC12232326; doi:10.1021/jacs.5c05460)
Supplement: Supplementary file 1 [file ja5c05460_si_001.pdf]

Supporting Information

Autonomous dynamic control of crown ether cargo release from [2]rotaxane carriers in a piperidine oscillator

Kamil D. Petryczkiewicz, Johanan Kootstra, Maëlle Le Cacheux, Aleksei Tsygankov, Jan L. Sneep, Matthijs ter Harmsel, Syuzanna R. Harutyunyan*

Stratingh Institute for Chemistry, University of Groningen, Groningen, 9747 AG, the Netherlands.

Correspondence and requests for materials should be addressed to S.R.H. (e-mail: s.harutyunyan@rug.nl)

Table of contents

1. List of abbreviations 3
2. General experimental information 5
3. Substrates synthesis 7
4. Optimization of carrier synthesis 12
5. Gram-scale carrier synthesis 13
6. Temperature determination in NMR 16
7. Cargo release in batch 18
8. Influence of crown ether 21
9. Oscillatory cargo release 26
10. NMR spectra of isolated compounds 32
10. References 40

1. List of abbreviations

24C8 - 24-Crown-8 ether

AcCl - Acetyl chloride

db24C8 - Dibenzo-24-crown-8 ether

DBF - Dibenzofulvene

DCM – Dichloromethane

DMF – Dimethylformamide

DMSO - Dimethyl sulfoxide

ESI- Electrospray ionization

Et_2O - Diethyl ether

Et_3N – Triethylamine

EtOAc - Ethyl acetate

Fmoc-pip - *N*-[(9H-Fluoren-9-ylmethoxy)carbonyl]piperidine

Fmoc-R - Fluorenylmethyloxycarbonyl group

FTIR - Fourier-transformed infrared spectroscopy

GC - Gas chromatography

GC-FID - Gas chromatography-flame ionization detector

MeOH - Methanol

NMR - Nuclear magnetic resonance

Np-Ac - 4-Nitrophenyl acetate

Np-Prop - 4-Nitrophenyl propionate

PhOAc - Phenyl acetate

Pip-Ac - *N*-acetylpiperidine

Pip-Prop - *N*-propanoylpiperidine

PTFE - Teflon

R-Np - 4-Nitrophenyl group

R-OSu - *N*-Hydroxysuccinimide group

TBBA - 3,5-Di-*tert*-butylbenzylamine

TBME - *tert*-Butyl methyl ether

TFBA - 3,5-Bis(trifluoromethyl)benzylamine

TFBA-Ac - *N*-3,5-Bis(trifluoromethyl)benzylacetamide

TFBA-Prop - *N*-3,5-Bis(trifluoromethyl)benzylpropanamide

THF - Tetrahydrofurane

TMB - 1,3,5-Trimethoxybenzene

2. General experimental information

Flash column chromatography was performed using Merck 60 Å 230–400 mesh silica gel.

NMR spectroscopic data were collected on a Varian MercuryPlus (^{1}H at 400 MHz; ^{13}C at 101 MHz) equipped with a 400 AutoSw probe, a Varian 400MR (^{1}H at 400 MHz; ^{13}C at 101 MHz) equipped with a OneNMR probe, a Varian Inova 500 (^{1}H at 500 MHz) equipped with a Varian 5 mm PFG SW probe and a Varian MercuryPlus (^{1}H at 300 MHz) equipped with a 300 Autosw probe. Chemical shifts are reported in parts per million (ppm) relative to residual solvent peak ($CDCl_3$, ^{1}H: 7.26 ppm; ^{13}C: 77.16 ppm and DMSO-d_6, ^{1}H: 2.50 ppm,^{13}C: 39.53 ppm). Coupling constants are reported in Hertz (Hz). Multiplicity is reported with the usual abbreviations (s: singlet, d: doublet, t: triplet, td: triplet of doublets, q: quadruplet, m: multiplet).

Exact mass spectra for compound characterization were recorded on the Thermo Scientific Orbitrap Exploris 480 with ESI ionization.

Batch reactions (single pulse experiments) and acetylation of TFBA by PhOAc were monitored by ^{1}H NMR. The obtained spectra were analyzed with *MestreNova*, phased and baseline corrected. Artifacts were removed by setting drift correction to 1%. The integration of relevant peaks was performed using the *Integral Graph* function. Concentration of reaction components was determined in reference to a TMB internal standard peak – 6.14 ppm (s, 3H) and by integration of relevant peaks: TFBA – 8.53 ppm (s, 1H), TFBA-Ac – 7.97 ppm (m, 3H), phenol – 6.80 ppm (m, 3H), DBF – 6.27 ppm (s, 2H), db24C8 – 4.07 ppm (m, 8H), 24C8 – 3.56 ppm (s, 32H), Pip-Ac – 1.97 ppm (s, 3H).

For experiments under flow conditions, reactions were performed using a dual-channel syringe pump (in-flow, New Era SyringeTwo), a single-channel syringe pump (out-flow, New Era SyringeOne), and Labm8 M8003.1 pump with the corresponding control unit. To connect syringes with the reactor, PTFE tubing (0.56 mm ID x 1.07 mm OD) was used.

Sample collection and quenching for flow experiments were conducted using a modified GC-PAL Autosampler setup operated by a *Python* script through serial communication.[1] Samples were quenched by injection into Np-Prop solution in EtOAc to deactivate any free amines. The concentration of reaction components was quantified via GC-FID measurements against a TMB internal standard and calculated using previously prepared calibration curves. GC-FID data was collected on an Agilent Technologies 7890 A GC System with a HP-5MS column. Obtained data was processed by converting GC data files to CSV file and subsequently plotted, integrated and combined using a *Python* script, which can be accessed on GitHub (https://github.com/RenzeSneep/GC-data-analysis).

For monitoring acetylation of TFBA by 4-nitrophenyl acetate (Np-Ac) and control batch reactions, *in situ* FTIR reaction analysis was used. Monitoring was performed using a Mettler-Toledo ReactIRTM 700 instrument fitted with a DiComp (diamond) probe, an AgX Fiber Conduit, and a liquid N_2-cooled MCT detector. Spectra were recorded with 8 cm^{-1} resolution. IR spectra were analyzed using *iC IR 7.1*.

Unless otherwise indicated, reagents and substrates were purchased from commercial sources and used as received. Chemicals were purchased from Sigma-Aldrich, TCI and BLDpharm. Solvents not required to be dry were purchased as technical grade or spec-grade and used as received. Known compounds were characterized by ^{1}H NMR and compared to the reported data. New compounds were characterized by ^{1}H NMR, ^{13}C NMR and HRMS.

Note about temperature control

In our previous work, the reaction temperature was monitored by an *in situ* FTIR probe equipped with a thermometer. We found that this probe was faulty, and the actual reaction temperature used for flow experiments was 68 °C instead of 60 °C as in other experiments. Thus, we decided to conduct flow experiments also at 68 °C. To reliably control the temperature of reactions monitored by *in situ* FTIR or GC-FID, we used an external temperature probe Voltcraft PL-120 T1 equipped with a Type K thermocouple. In all experiments, this thermocouple was used to determine the internal reaction temperature, while a secondary probe connected to an IKA stirrer controlled the bath temperature. To control the temperature of reactions monitored by ^{1}H NMR, a protocol was developed as described in Section 5.

Note about carrier 1 solubility

No experiments with carrier 1 concentration higher than 0.15 M were performed as larger amounts of the carrier could not be solubilized even after sonicating the solution at 60 °C.

3. Substrates synthesis

3,5-Di-*t*-butylbenzylamine (TBBA)

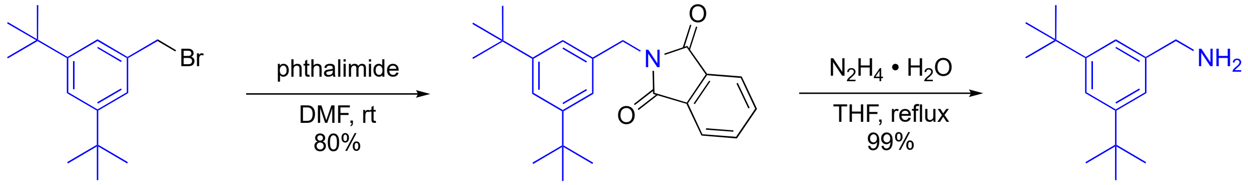

Scheme S1. Two-step synthesis of TBBA from 3,5-di-*t*-butylbenzyl bromide.

The procedure was adapted from the literature.[2]

3,5-Di-*t*-butylbenzyl bromide (5.03 g, 17.8 mmol, 1.0 equiv.), phthalimide (3.38 g, 22.9 mmol, 1.3 equiv.) and K_2CO_3 (3.66 g, 26.5 mmol, 1.5 equiv.) were dissolved in DMF (70 mL). The reaction mixture was stirred for 24 h at room temperature. Et_2O (100 mL) was added and the resulting solution was filtered, and washed with water (200 mL) and brine (100 mL). The organic fraction was collected, dried over $MgSO_4$, filtered, and concentrated *in vacuo*. The resulting white solid of the *N*-alkylphthalimide (4.91 g, 14.1 mmol, 1.0 equiv.) was dissolved in THF (80 mL). Hydrazine monohydrate (4.1 mL, 84 mmol, 6.0 equiv.) was added dropwise to the stirring solution. The mixture was kept at reflux for 3 h, during which a white precipitate appeared. The solid was dissolved by adding water (100 mL) and the solution was extracted with TBME (2 x 100 mL). The combined organic layers were washed with brine (100 mL), dried over $MgSO_4$, filtered and concentrated *in vacuo*. The product was obtained as a white solid (3.06 g, 79% over two steps).

^{1}H NMR (400 MHz, $CDCl_3$) δ 7.34 (s, 1H), 7.17 (s, 2H), 3.87 (s, 2H), 1.65 (s, 2H), 1.35 (s, 18H).

^{13}C NMR (101 MHz, $CDCl_3$) δ 151.1, 142.6, 121.4, 121.1, 47.3, 35.0, 31.6.

24-Crown-8 ether (24C8)

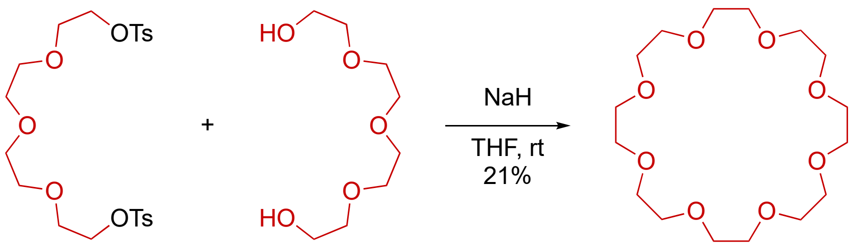

Scheme S2. Synthesis of 24C8 from tetraethylene glycol ditosylate and tetraethylene glycol.

The procedure was adapted from literature.[3]

NaH (6.36 g, 158.9 mmol, 3.23 equiv., 60% dispersion) was washed with *n*-hexane to remove the protecting oil. The washed NaH was suspended in THF (200 mL) under N_2 atmosphere. A solution of tetraethylene glycol (9.55 g, 49.2 mmol, 1.0 equiv.) and tetraethylene glycol ditosylate (24.72 g, 49.2 mmol, 1.0 equiv.) in THF (800 mL) was prepared. The solution was added dropwise to the stirring suspension of NaH using a peristaltic pump (20 mL/h). The mixture was left to stir for 96 h at room temperature. The unreacted NaH was destroyed by addition of water (2 mL). Celite (10 g) was added and the suspension was filtered and concentrated *in vacuo*. The residue was extracted with boiling *n*-hexane (4 x 150 mL) and the combined extracts were concentrated *in vacuo*. The resulting yellow liquid was dissolved in a minimal amount of acetonitrile, filtered and stored in the freezer (-20°C) overnight. The formed colorless crystals were filtered and washed with ice-cold acetonitrile. The crystallization procedure was repeated twice and the combined crystals were dried *in vacuo* yielding the product as a colorless liquid (3.60 g, 21%).

^{1}H NMR (400 MHz, $CDCl_3$): δ 3.68 (s, 32H).

Fmoc 4-nitrophenol ester (Fmoc-Np)

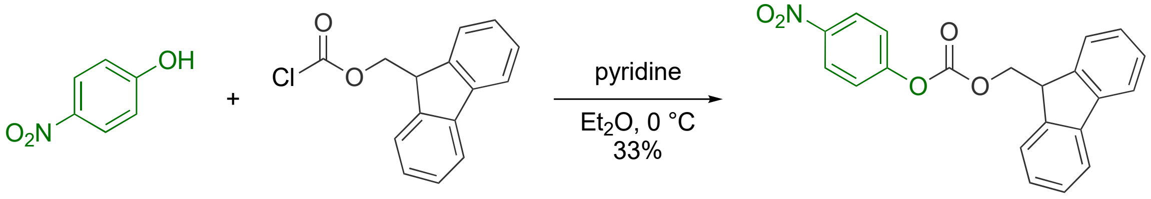

Scheme S3. Synthesis of Fmoc-Np from 4-nitrophenol and Fmoc-Cl.

The procedure was adapted from literature.[4]

Fmoc-Cl (5.00 g, 19.3 mmol, 1.0 equiv.) was dissolved in Et_2O (25 mL) and cooled with an ice bath. A solution of 4-nitrophenol (2.69 g, 19.3 mmol, 1.0 equiv.) and pyridine (7.8 mL, 97 mmol, 5.0 equiv.) in Et_2O (25 mL) was added dropwise using a syringe pump (1 mL/min). The solution was stirred for 2 h and a white precipitate appeared. The solution was filtered and the solid was washed with Et_2O. The organics were washed with $HCl_{(aq)}$ (1 M, 2 x 100 mL), water (100 mL), brine (100 mL), dried over $MgSO_4$, and filtered. The organic layers were collected and evaporated *in vacuo*. The product was purified by flash chromatography (SiO_2, 1:19 EtOAc/pentane to 1:9 EtOAc/pentane) yielding a yellow solid (2.42 g, 33%).

^{1}H NMR (400 MHz, $CDCl_3$): δ 8.27 (d, J = 9.1 Hz, 2H), 7.80 (d, J = 7.6 Hz, 2H), 7.64 (d, J = 7.4 Hz, 2H), 7.45 (t, J = 7.5 Hz, 2H), 7.35 (t, J = 7.7 Hz, 4H), 4.61 (d, J = 7.0 Hz, 2H), 4.34 (t, J = 7.0 Hz, 1H).

^{13}C NMR (101 MHz, $CDCl_3$): δ 155.6, 152.5, 145.5, 143.0, 141.5, 128.3, 127.4, 125.4, 125.1, 121.9, 120.4, 71.0, 46.7.

Fmoc piperidine (Fmoc-pip)

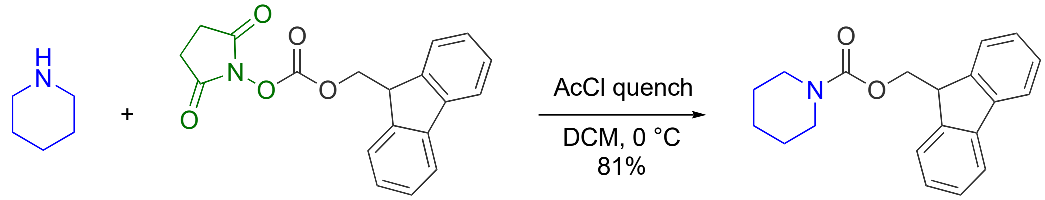

Scheme S4. Synthesis of Fmoc-pip from piperidine and *N*-hydroxysuccinimide ester.

The procedure was adapted from literature.[5]

Fmoc *N*-hydroxysuccinimide ester (14.9 g, 44 mmol, 1.1 equiv.) was dissolved in DCM (160 mL). The solution was cooled to 0 °C using an ice bath. Piperidine (4.0 mL, 40 mmol, 1.0 eq) was added over 20 minutes using a syringe pump. The reaction was left to stir for 2 hours and was quenched with acetyl chloride (2.9 mL, 40 mmol, 1.0 eq). After 30 minutes the reaction mixture was washed with water (150 mL) and brine (150 mL). Organics were dried over $MgSO_4$ and evaporated *in vacuo*. The crude was purified using flash chromatography (1:4 EtOAc/pentane) affording the product as a white powder (10.0 g, 81%).

^{1}H NMR (400 MHz, $CDCl_3$): δ 7.77 (d, J = 7.6 Hz, 2H), 7.59 (d, J = 7.5 Hz, 2H), 7.40 (t, J = 7.5 Hz, 2H), 7.32 (td, J = 7.4, 1.2 Hz, 2H), 4.40 (d, J = 7.0 Hz, 2H), 4.26 (t, J = 7.0 Hz, 1H), 3.48 – 3.42 (m, 4H), 1.73 – 1.37 (m, 6H).

4-Nitrophenyl propionate (Np-Prop)

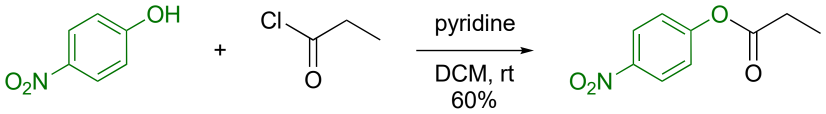

Scheme S5. Synthesis of Np-Prop from 4-nitrophenol and propionyl chloride.

The procedure was adapted from literature.[6]

4-Nitrophenol (23.15 g, 166 mmol, 1.1 equiv.) and pyridine (19.5 mL, 242 mmol, 1.6 equiv.) were dissolved in DCM (100 mL). Propionyl chloride (13.2 mL, 151 mmol, 1.0 equiv.) was added dropwise to the reaction mixture and left to stir for 3 h. The mixture was washed with water (100 mL), $HCl_{(aq)}$ (2 M, 100 mL), and brine (100 mL). The organic phase was dried over $MgSO_4$ and concentrated *in vacuo*. The crude product was recrystallized from Et_2O giving the final product (17.8 g, 60%).

^1H NMR (400 MHz, $CDCl_3$): δ 8.27 (d, J = 9.2 Hz, 2H), 7.28 (d, J = 9.2 Hz, 2H), 2.64 (q, J = 7.5 Hz, 2H), 1.28 (t, J = 7.5 Hz, 3H).

4. Optimization of carrier synthesis

Table S1. Optimization of carrier synthesis[a]

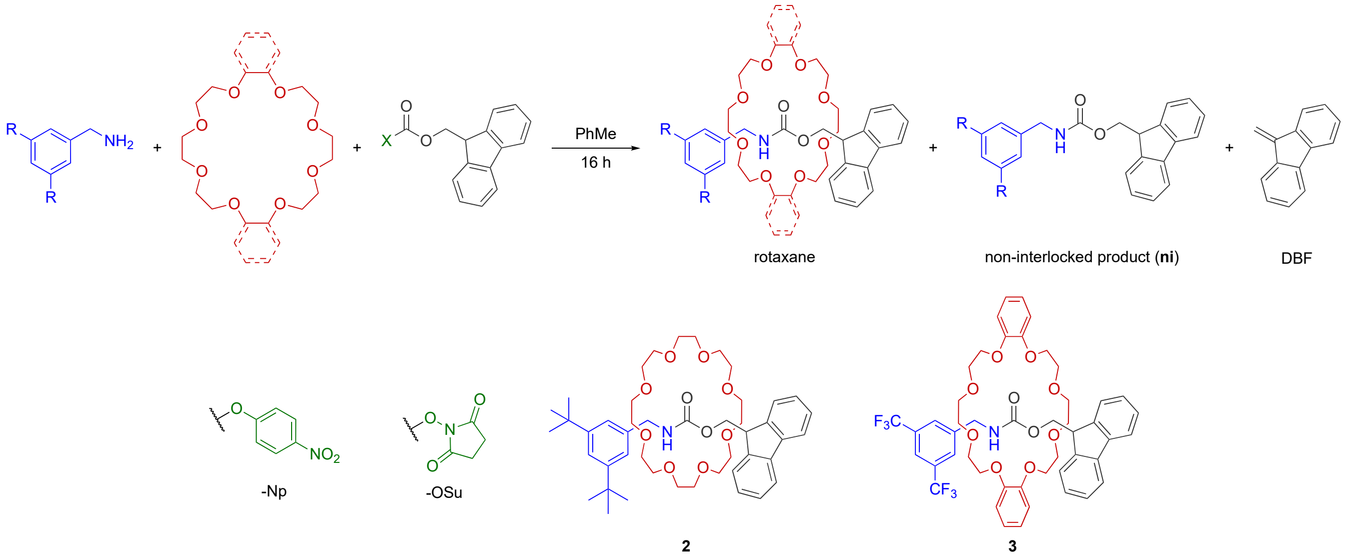

Entry	X	T [°C]	Solvent	Conversion (Fmoc-X) [%][b]		Yield [%][b]		Products ratio[b]	
				2	3	2	3	**2**:**2ni**:DBF	**3**:**3ni**:DBF
1[c]	-Cl	rt	PhMe	>99	>99	11	28	11:89:1	28:61:11
2	-OSu	rt	PhMe	>99	88	0	7	0:95:5	7:81:0
3	-OSu	rt	DCM	>99	86	0	2	0:91:9	2:84:0
4[c]	-Cl	0	PhMe	>99	>99	17	29	17:82:1	29:63:8
5[c]	-Np	0	PhMe	>99	>99	65	69	65:27:8	69:17:14

[a]Reaction conditions: 0.14 M benzylamine, crown ether (1 eq.), Fmoc-X (1 eq.) at T for 16 h. [b]Determined by ^{1}H NMR of reaction crude. [c]Et_3N (2 eq.) was added to neutralize the acidic side product (HCl or NpH).

In general, we observed full conversion of the Fmoc substrate, except for the reaction of Fmoc-OSu with TFBA exemplifying the higher reactivity of TBBA. Moreover, analysis of the crude reaction mixture revealed the occurrence of two side reactions: elimination to DBF and non-templated acylation, with the latter occurring most readily with Fmoc-OSu. Perhaps this is due to steric repulsion in the transition state between the carbonyl oxygen of the leaving group and the crown ether. In contrast, the use of Fmoc-Np promoted the rotaxane formation even more than Fmoc-Cl. We hypothesize that the direct reaction with Fmoc-Cl is too fast to be outcompeted by the entropically unfavorable templated reaction. For **2** this occurs because TBBA is more reactive than TFBA, while in the case of **3**, the benzene rings in db24C8 reduce the hydrogen-bonding capabilities of the oxygen atoms compared to 24C8.[7] This entropic effect can be influenced by lowering the temperature and promoting the kinetic rotaxane product, but it is not sufficient to reverse the selectivity. However, when Fmoc-Np is used, acylation is slower, and the crown ether can provide sufficient transition state stabilization to promote the rotaxane formation.

5. Gram-scale carrier synthesis

General procedure for carrier synthesis (A)

The procedure was adapted from the literature and optimized according to Table S1.[7]

Benzylamine (1.0 equiv.) and crown ether (1.0 equiv.) were dissolved in toluene. The solution was cooled to 0 °C with an ice bath. Fmoc derivative (1.0 equiv.) and Et_3N (2.0 equiv.) were dissolved in toluene and added dropwise (1 mL/h) to the reaction mixture. The solution was stirred for 16 h and concentrated *in vacuo*. The product was purified by flash chromatography. To remove residual solvent, some Et_2O was added, and the solution was concentrated *in vacuo.*

Carrier **1**

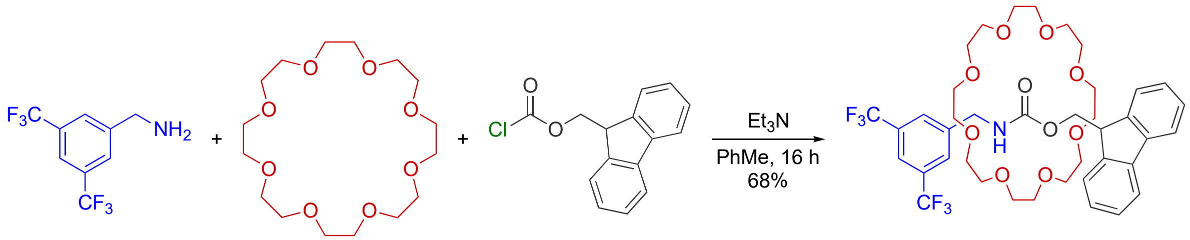

Scheme S6. Synthesis of Fmoc-TFBA 24C8 amide-axel [2]rotaxane from TFBA, Fmoc-Cl and 24C8.

The product was synthesized using the general procedure A with TFBA (2.12 g, 8.72 mmol, 1.0 equiv.), and previously synthesized 24C8 (3.07 g, 8.72 mmol, 1.0 equiv.) in toluene (40 mL) and Fmoc-Cl (2.26 g, 8.72 mmol, 1.0 equiv.) and Et_3N (2.4 mL, 17.44 mmol, 2.0 equiv.) in toluene (30 mL). Flash chromatography (SiO_2, 30:1 DCM/MeOH) afforded the product as a white solid (4.87 g, 68%).

As reported previously, carrier 1 exists as two conformers with ~1:8 ratio.[7] Only signals for the major conformer are reported here.

^{1}H NMR (400 MHz, $CDCl_3$): δ 8.55 (s, 2H), 7.75 – 7.68 (m, 4H), 7.67 (s, 1H), 7.36 (t, J = 7.4 Hz, 2H), 7.26 (td, J = 7.5, 1.2 Hz, 2H), 6.73 (t, J = 4.2 Hz, 1H), 4.70 (d, J = 4.2 Hz, 2H), 4.54 (d, J = 6.0 Hz, 2H), 4.17 (t, J = 6.0 Hz, 1H), 3.45 – 3.34 (m, 16H), 3.23 – 3.13 (m, 16H).

^{13}C NMR (101 MHz, $CDCl_3$): δ 156.4, 144.7, 141.8, 141.6, 133.5, 129.1 (q, J = 272.3), 127.5, 127.0, 125.3, 124.3 (q, J = 32.3), 119.8, 119.5 (t, J = 3.7 Hz), 70.6, 65.1, 47.8, 44.6.

Carrier **2**

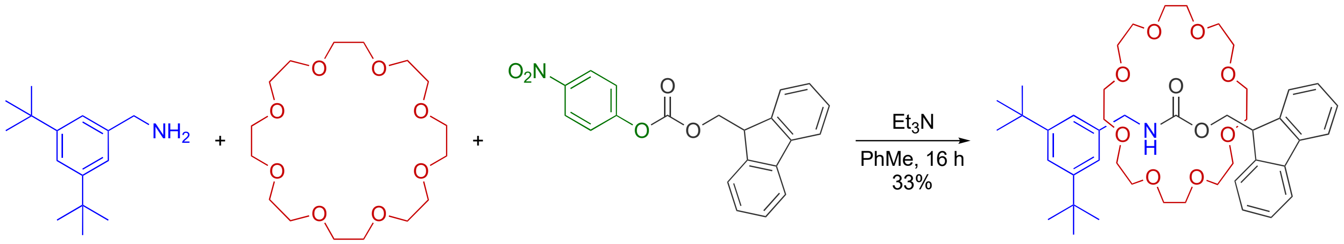

Scheme S7. Synthesis of Fmoc-TBBA 24C8 amide-axel [2]rotaxane from TBBA, Fmoc-Np and 24C8.

The product was synthesized using the general procedure A with previously synthesized TBBA (1.25 g, 5.68 mmol, 1.0 equiv.), and previously synthesized 24C8 (2.0 g, 5.68 mmol, 1.0 equiv.) in toluene (24 mL) and Fmoc-Np (2.05 g, 5.68 mmol, 1.0 equiv.) and Et_3N (1.6 mL, 11.35 mmol, 2.0 equiv.) in toluene (16 mL). Flash chromatography (SiO_2, 1:1 EtOAc/pentane) afforded the product as a white solid (1.49 g, 33%).

^{1}H NMR (400 MHz, $CDCl_3$): δ 7.78 (d, J = 7.5 Hz, 2H), 7.73 (d, J = 7.5 Hz, 1H), 7.67 (s, 2H), 7.35 (t, J = 7.4 Hz, 2H), 7.27 (t, J = 7.4 Hz, 2H), 7.18 (s, 1H), 6.88 (s, 1H), 4.57 (s, 2H), 4.50 (d, J = 6.5 Hz, 2H), 4.15 (t, J = 6.5 Hz, 1H), 3.51 – 3.42 (m, 16H), 3.31 – 3.18 (m, 16H), 1.30 (s, 18H).

^{13}C NMR (101 MHz, $CDCl_3$): δ 156.5, 148.6, 144.9, 141.5, 137.3, 127.4, 127.3, 127.1, 125.7, 119.7, 119.6, 70.4, 65.1, 53.6, 47.9, 46.1, 35.0, 31.8.

HRMS (ESI$^+$) m/z = 794.4832 $[M+H]^+$, calculated for $C_{46}H_{67}NO_{10}$: 794.4838

Carrier **3**

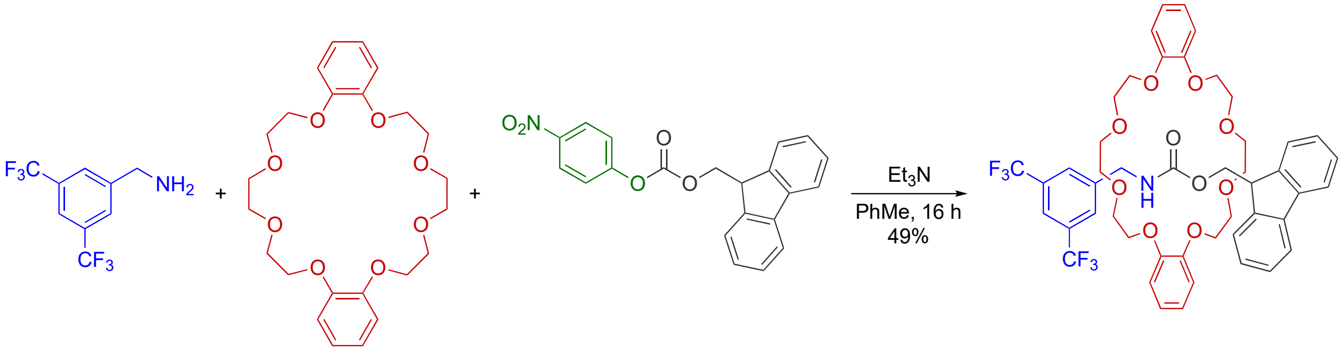

Scheme S8. Synthesis of Fmoc-TFBA db24C8 amide-axel [2]rotaxane from TFBA, Fmoc-Np and db24C8.

The product was synthesized using the general procedure A with TFBA (1.81 g, 7.46 mmol, 1.0 equiv.), and db24C8 (3.35 g, 7.46 mmol, 1.0 equiv.) in toluene (65 mL) and Fmoc-Np (2.70 g, 7.46 mmol, 1.0 equiv.) and Et_3N (2.1 mL, 14.92 mmol, 2.0 equiv.) in toluene (35 mL). Flash chromatography (SiO_2, 1:1 EtOAc/toluene) afforded the product as a white solid (3.35 g, 49%).

In $CDCl_3$ and at ambient temperature, carrier **3** exists as two conformers with ~1:3 ratio. Only signals for the major conformer are reported here.

^{1}H NMR (400 MHz, $CDCl_3$): δ 8.34 (s, 2H), 7.71 – 7.59 (m, 4H), 7.32 (t, J = 7.4 Hz, 2H), 7.17 (t, J = 7.4 Hz, 2H), 7.10 (s, 1H), 6.85 (t, J = 4.4 Hz, 1H), 6.72 – 6.67 (m, 4H), 6.59 – 6.53 (m, 4H), 4.87 (d, J = 3.9 Hz, 2H), 4.23 (d, J = 6.8 Hz, 2H), 4.06 – 3.86 (m, 12H), 3.86 – 3.79 (m, 4H), 3.68 – 3.54 (m, 8H).

^{13}C NMR (101 MHz, $CDCl_3$): δ 156.0, 147.7, 144.6, 142.0, 141.5, 131.7, 128.7 (q, J = 32.6 Hz), 127.5, 126.9, 126.2, 125.4, 122.7, 120.2, 119.8, 118.9, 116.0, 111.2, 70.6, 69.8, 67.8, 65.5, 47.5, 44.1.

HRMS (ESI$^+$) m/z = 914.3337 $[M+H]^+$, calculated for $C_{48}H_{49}F_6NO_{10}$: 914.3333

6. Temperature determination in NMR

During our research, we encountered problems with reproducing some of the batch cargo release results. Even when performing *duplo* experiments from the same stock solution, different lag phase lengths and pulse widths were sometimes observed. This indicated that the temperature in the NMR machine varied between experiments, and the temperature probe of the machine was not reliable. To ensure proper temperature control, we prepared a calibration curve relating sample temperature and ^{1}H NMR chemical shift of the sample components. Since we conducted our experiments in DMSO-*d$_6$* with TMB as the internal standard, we chose for the calibration the residual DMSO signal (2.50 ppm) and the TMB methyl group signal (3.72 ppm). Additionally, because PhOAc (slow inhibitor) is present in the reaction mixture in large quantities during batch experiments, PhOAc was also added to the sample to match the experimental conditions.

To obtain the chemical shifts of relevant peaks, spectra were analyzed with *MestreNova*, phased, baseline corrected, and artifacts were removed by setting drift correction to 1%. Then, chemical shifts were determined using the *Maximum Peak Position* function.

<u>Temperature calibration curve for NMR experiments</u>

Using volumetric glassware, a solution of TMB (8.4 mg, 0.05 mmol, 1.0 equiv.) and PhOAc (127 µL, 1.0 mmol, 20 equiv.) in DMSO-*d$_6$* (2 mL) was prepared. The solution (600 µL) was transferred into an NMR tube. The machine temperature was set to 50 °C and after letting the temperature equilibrate for 5 minutes, a ^{1}H NMR spectrum of an ethylene glycol chemical standard sample was measured. Using *VnmrJ* and the built-in *tempcal('g')* command, the actual temperature of the ethylene glycol sample was recorded from the obtained ^{1}H NMR spectrum. The TMB sample was inserted into the machine, left to equilibrate for 5 minutes and the ^{1}H NMR spectrum was measured. The machine temperature was increased by 2 °C and the procedure was repeated until reaching 70 °C (Figure S1). Using the obtained spectra, the difference between the DMSO and TMB methyl group chemical shifts was determined. With the actual temperature data and the chemical shifts, a line of best fit was found using *Microsoft Excel* (Equation S1, Figure S2)

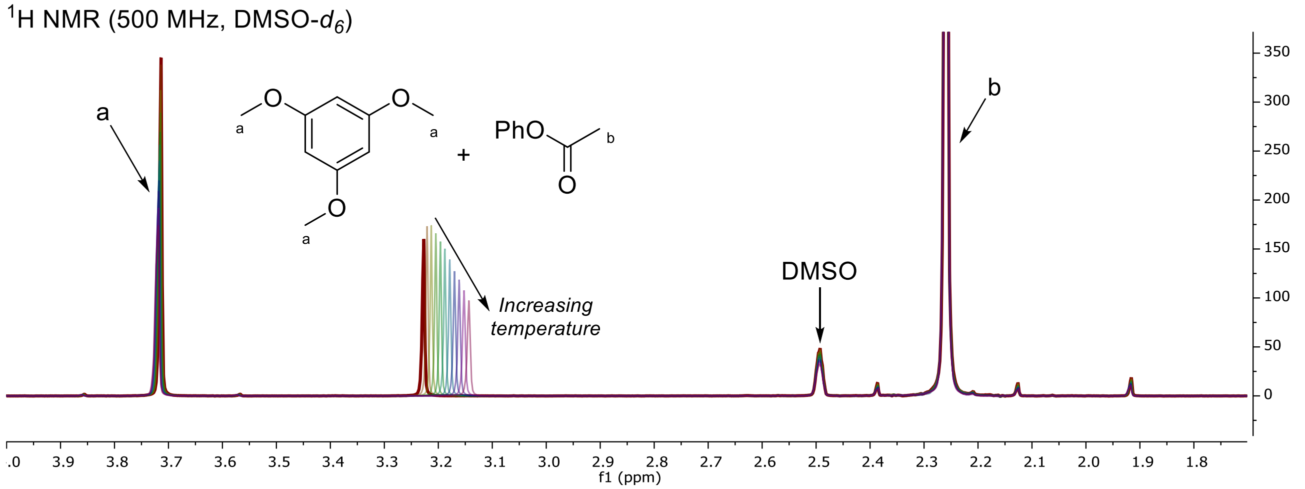

Figure S1. Zoom-in of the set of ^{1}H NMR spectra obtained during temperature calibration for machine temperature 50 °C – 70 °C.

$$T = 2713 \times \delta_{TMB}^{DMSO} - 3264$$

Equation S1. Equation of the line of best fit for the temperature calibration. T is the temperature in degrees Celsius (°C) and δ_{TMB}^{DMSO} is the difference between the chemical shift of the TMB methyl group and DMSO expressed in parts per million (ppm).

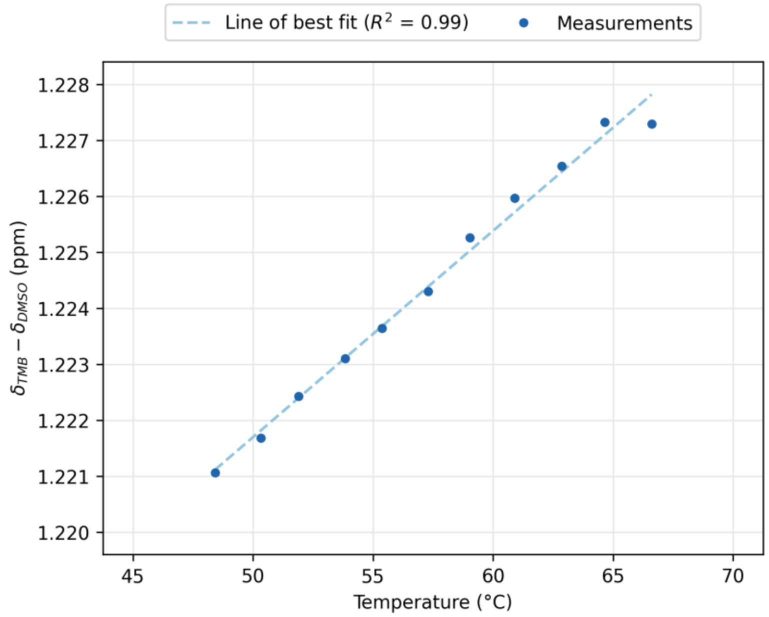

Figure S2. Temperature calibration curve for NMR measurements. Actual sample temperature was determined using an ethylene glycol standard. The change of chemical shift is reported as the chemical shift of the TMB methoxy group with respect to DMSO. The equation of the best-fit line is shown in Equation S1.

General procedure for determining sample temperature in NMR (B)

The NMR machine was set to the target temperature. The sample was placed in the machine and the temperature was allowed to equilibrate for 5 minutes. A ^{1}H NMR spectrum of the sample was measured and the difference between the DMSO and TMB methyl group chemical shifts was determined. Using the temperature calibration curve (Figure S2, equation S1), sample temperature was calculated. If necessary, the machine temperature was adjusted and the temperature was measured again until the desired conditions were reached.

7. Cargo release in batch

General protocol for batch cargo release in NMR (C)

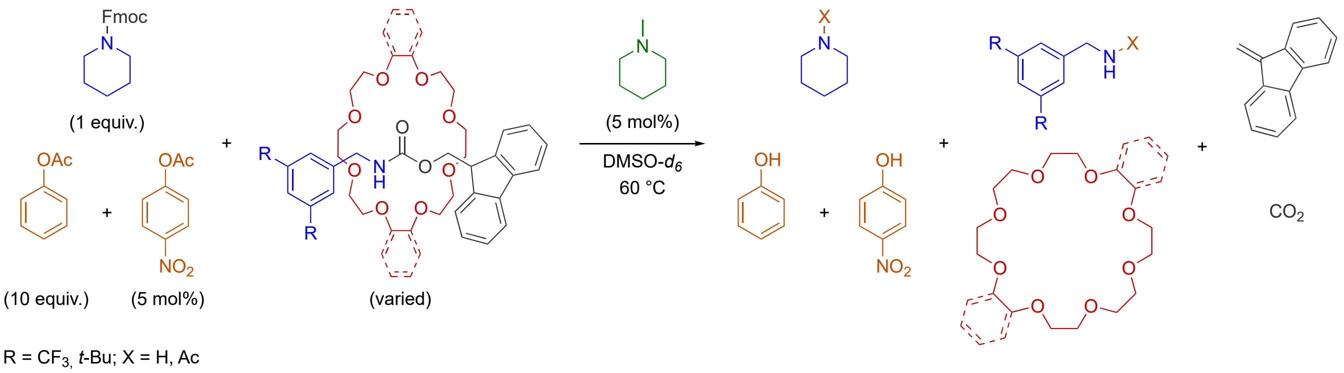

Scheme S9. General reaction scheme for batch cargo release experiments with carriers **1**-**3**.

Fmoc-pip (30.74 mg, 0.10 mmol, 1.0 equiv.), PhOAc (127 µL, 1.0 mmol, 10 equiv.), Np-Ac (0.91 mg, 0.05 mmol, 0.05 equiv.), TMB (8.41 mg, 0.05 mmol, 0.50 equiv.) and carrier (0.0 equiv. – 3.0 equiv.) were added to a volumetric flask. The flask was filled with DMSO-d_6 to a total volume of 2 mL (reagent stock). *N*-methylpiperidine (61 µL, 0.50 mmol, 5.0 equiv.) was added to a separate volumetric flask. The flask was filled with DMSO-d_6 to a total volume of 1 mL (trigger stock). Reagent stock (600 µL) was transferred to an NMR tube and placed in the NMR spectrometer. Temperature was adjusted to 60 °C using the general procedure B. Trigger stock (3 µL) was added (t = 0 min) and the tube was sealed with a perforated cap to allow CO_2 release. The reaction was monitored by ^{1}H NMR (Figure S3) for 100 minutes, taking measurements every minute with 40 second relaxation time, while a spectrum taken during the temperature adjustment was used as the t = 0 min measurement. Concentration of relevant species was determined using TMB as internal standard. Using the reaction mass balance, piperidine concentration was calculated at every time point.

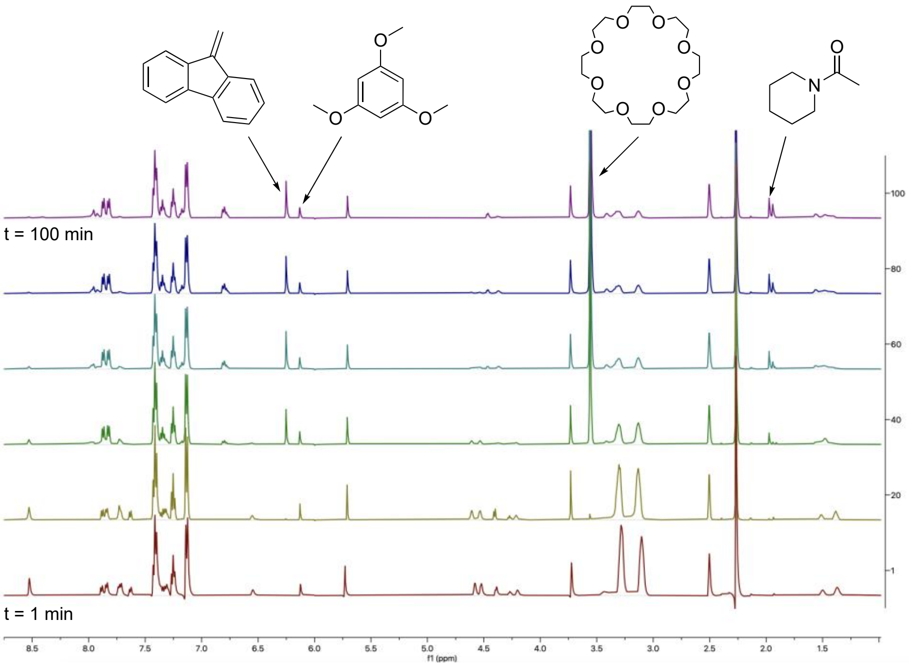

Figure S3. Zoom-in of an example NMR spectra from a batch cargo release experiment with carrier **1** (here: 1.0 equiv.). Signals used for determining concentration are marked with the corresponding compounds.

Batch cargo release for varied carrier **1** loading

Following the general procedure C, batch cargo release experiments were performed using carrier **1** (0.0 equiv. – 3.0 equiv.). Experiments were performed *in duplo*. Concentrations of DBF, Pip-Ac, 24C8 and piperidine were determined (Figure 4b-d, Figure S4). Maximum piperidine concentration and concentration of 24C8 after 100 minutes were extracted from the data. Results were reported as an average and its error from both trails.

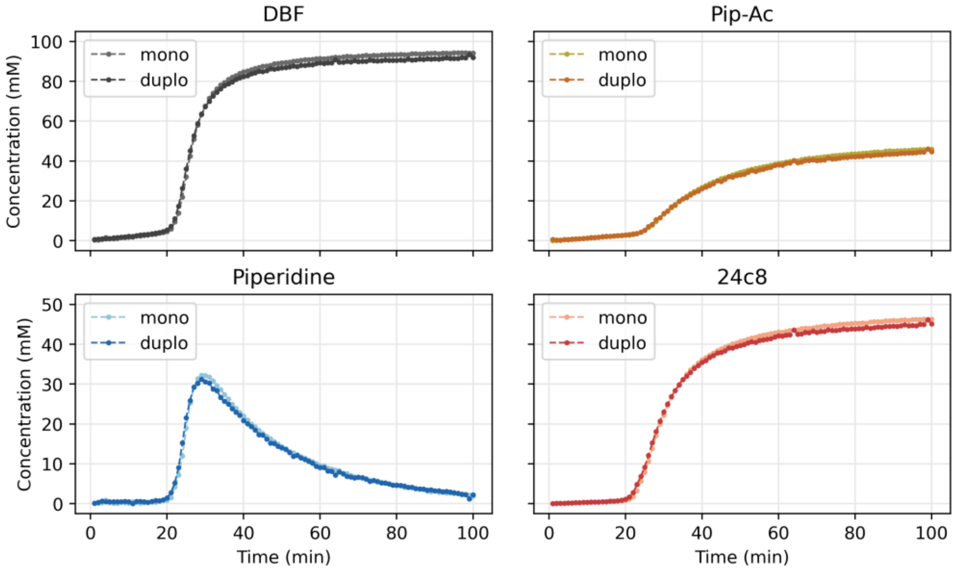

Figure S4. Example concentration plots from a batch cargo release experiment with carrier **1** (here: 1.0 equiv.).

Table S2. The influence of carrier loading on piperidine and cargo release[a]

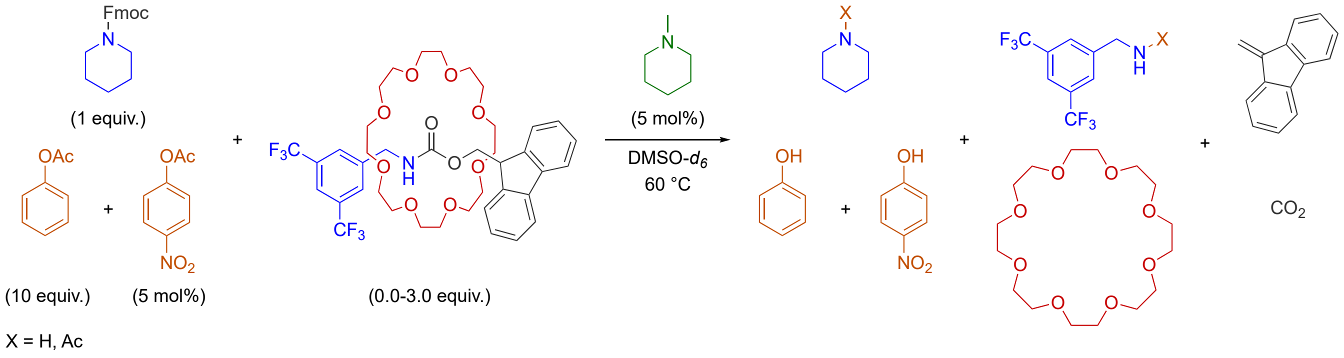

Entry	Carrier 1 [equiv.]	Pulse formed?	Pip amplitude [%][b]	Cargo release [%][c]
1	0.00	Yes	65	-
2	0.25	Yes	66	90
3	0.50	Yes	67	93
4	1.00	Yes	63	91
5	2.00	Yes	63	83
6	3.00	Yes	58	77

[a]Reaction conditions: 50 mM Fmoc-pip, carrier 1 (0.0-3.0 equiv.), trigger (5 mol%), fast inhibitor (5 mol%), slow inhibitor (10 equiv.) in DMSO-d_6 at 60 °C for 100 min. Monitored by ^{1}H NMR with TMB as an internal standard. Results are reported as means of *mono* and *duplo*. [b]The piperdine amplitude was calculated by taking the maximum piperidine concentration and dividing by the initial concentration of Fmoc-pip. [c]Cargo release was calculated by dividing the crown ether concentration at 100 min by the initial carrier concentration.

Batch cargo release for carrier **2**

Following the general procedure C, a batch cargo release experiment was performed using carrier **2** (39.68 mg, 0.05 mmol, 0.50 equiv.). The experiment was performed *in duplo*. The concentration of DBF, Pip-Ac, 24C8 and piperidine was determined. Maximum piperidine concentration and 24C8 concentration after 100 minutes were extracted from the data. Results are reported as an average and its error from both trails (Figure 5b).

Batch cargo release for carrier **3**

Following the general procedure C, a batch cargo release experiment was performed using carrier **3** (45.68 mg, 0.05 mmol, 0.50 equiv.). The experiment was performed *in duplo*. The concentration of DBF, Pip-Ac, db24C8 and piperidine was determined. Maximum piperidine concentration and db24C8 concentration after 100 minutes were extracted from the data. Results are reported as an average and its error from both trails (Figure 5b).

Table S3. The influence of carrier type on piperidine and cargo release[a]

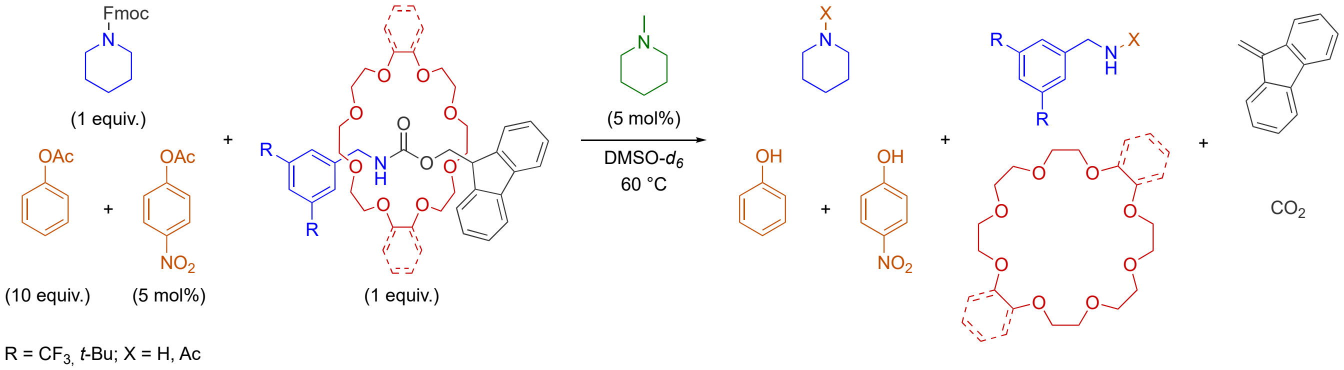

Entry	Carrier	Pulse formed?	Pip amplitude [%][b]	Cargo release [%][c]
1	1	Yes	67	93
2	2	Yes	65	83
3	3	Yes	62	89

[a]Reaction conditions: 50 mM Fmoc-pip, carrier (1.0 equiv.), trigger (5 mol%), fast inhibitor (5 mol%), slow inhibitor (10 equiv.) in DMSO-d_6 at 60 °C for 100 min. Monitored by ^{1}H NMR with TMB as an internal standard. Results are reported as means of *mono* and *duplo*. [b]The piperdine amplitude was calculated by taking the maximum piperidine concentration during the reaction and dividing by the initial concentration of Fmoc-pip. [c]Cargo release was calculated by dividing the crown ether concentration at 100 min by the initial carrier concentration.

8. Influence of crown ether

To investigate the influence of carrier decomposition on the oscillator we performed several control experiments: acetylation of TFBA by the inhibitors, acetylation of TFBA in the presence of 24C8 and a batch experiment without the carrier, but with addition of 24C8.

Acetylation of TFBA by PhOAc

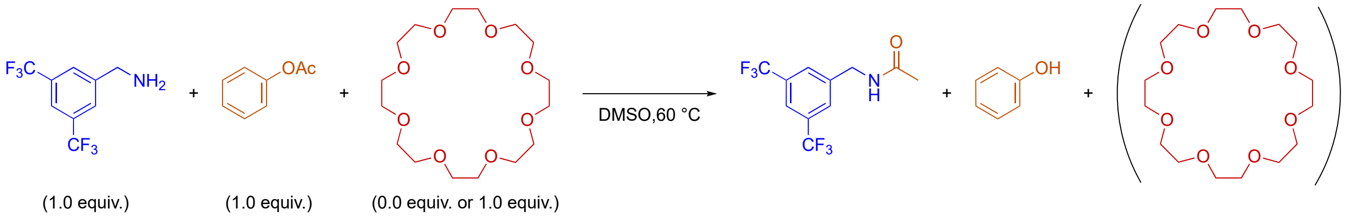

Scheme S10. TFBA acetylation by PhOAc (slow inhibitor) in the presence of 24C8.

In an NMR tube, TFBA (7.35 mg, 30.2 µmol, 1.0 equiv.) and TMB (4.94 mg, 29.4 µmol, 1.0 equiv.) were dissolved in DMSO-d_6 (600 µL). Reaction was initiated by addition of PhOAc (38 µL, 300 µmol, 10 equiv.) and monitored by ^{1}H NMR at 60 °C for 45 minutes with measurements taken every minute.

Following the same steps, TFBA (7.48 mg, 30.8 µmol, 1.0 equiv.), TMB (5.11 mg, 30.4 µmol, 1.0 equiv.) and 24C8 (5.11 mg, 30.4 µmol, 1.0 equiv.) were dissolved in DMSO-d_6 (600 µL). Reaction was initiated by addition of PhOAc (38 µL, 300 µmol, 10 equiv.) and monitored by ^{1}H NMR.

For both conditions, the formation of TFBA-Ac was observed. From the plots of concentration over time since PhOAc addition (Figure S5), a line of best fit was calculated in *Microsoft Excel*. The observed reaction rate constant was found to be the same for both experiments, indicating no influence of 24C8 (Table S4).

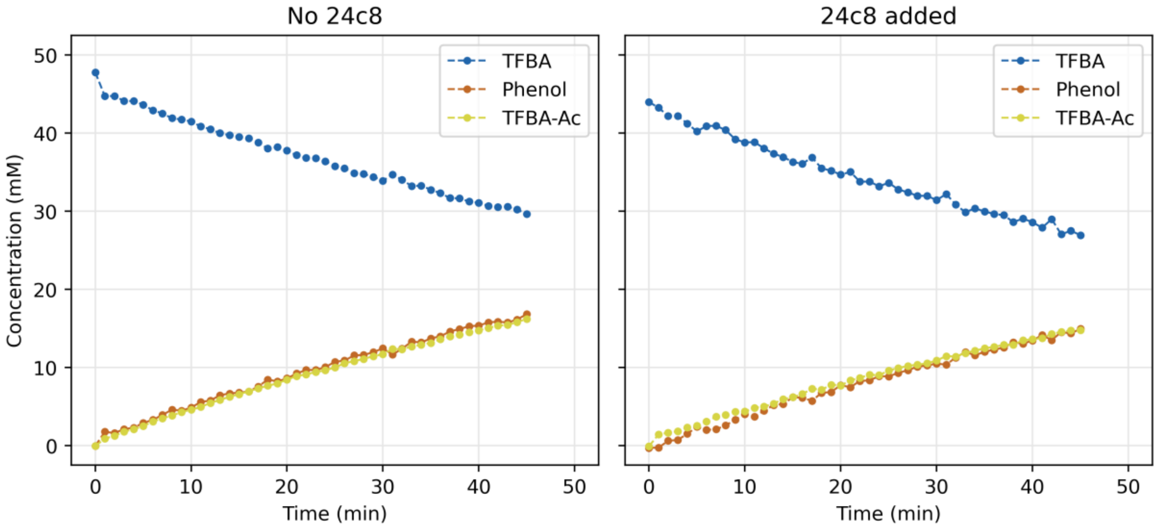

Figure S5. Concentration over time of TFBA (blue trace), phenol (orange trace) and TFBA-Ac (yellow trace) with and without addition of the 24C8 cargo.

Table S4. Acetylation of TFBA observed rate constant[a]

Experiment	Compound	Slope (M s^{-1})	k_{obs} (M^{-1} s^{-1})	R^2
No 24C8	TFBA	-5.92×10^{-6}	-2.35×10^{-4}	>0.99
	Phenol	5.88×10^{-6}	2.34×10^{-4}	>0.99
	TFBA-Ac	5.82×10^{-6}	2.31×10^{-4}	>0.99
24C8 added	TFBA	-6.02×10^{-6}	-2.34×10^{-4}	>0.99
	Phenol	5.59×10^{-6}	2.17×10^{-4}	>0.99
	TFBA-Ac	5.26×10^{-6}	2.04×10^{-4}	>0.99
Pip + PhOAc[b]	-	-	2.80×10^{-3}	-

[a]The rate constant was calculated from the slope of the concentration *v* time plot of reaction components. Irreversible first order kinetics with respect to TFBA and PhOAc were assumed. [b]The reference rate constant for the reaction of piperidine with PhOAc was taken as determined in our previous work.[4]

Acetylation of TFBA by Np-Ac

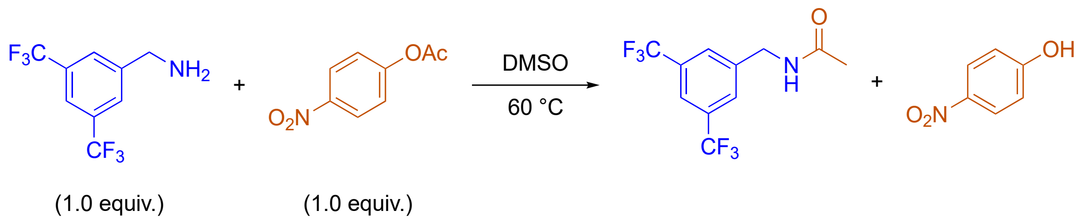

Scheme S11. TFBA acetylation by Np-Ac (fast inhibitor).

In a three-necked flask, Np-Ac (72.5 mg, 0.40 mmol, 1.0 equiv.) was dissolved in a DMSO (4 mL). The flask was placed in an oil bath and the temperature was adjusted to 60 °C and monitored using an external temperature probe. TFBA (97.3 mg, 0.40 mmol, 1.0 equiv.) was added and the reaction was monitored using an *in situ* FTIR probe for 15 minutes taking measurements every 5 seconds. The absorbance over time of TFBA acetate (TFBA-Ac, area: 1688 cm^{-1} to 1660 cm^{-1}, baseline: 1690 cm^{-1} to 1657 cm^{-1}) was determined using the *two-point baseline* integration function in iC IR 7.1 software (Figure S6 and S7).

The experiment revealed that the TFBA released during carrier cleavage has the potential to disrupt the oscillatory system due to its rapid reaction with the fast inhibitor.

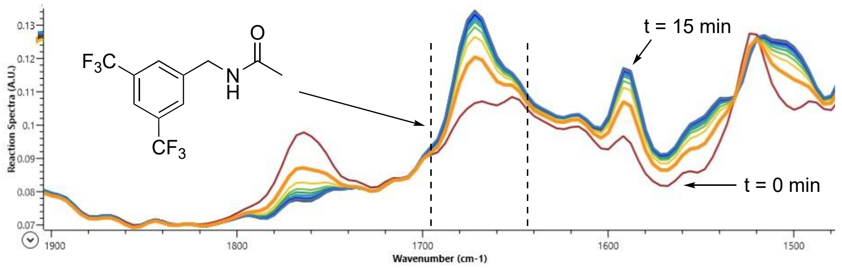

Figure S6. Zoom-in of FTIR spectra obtained while monitoring the reaction of TFBA with Np-Ac. Spectra range from 0 minutes (red) to 15 minutes (blue) of reaction time. The monitored band corresponding to TFBA-Ac is marked with dashed lines.

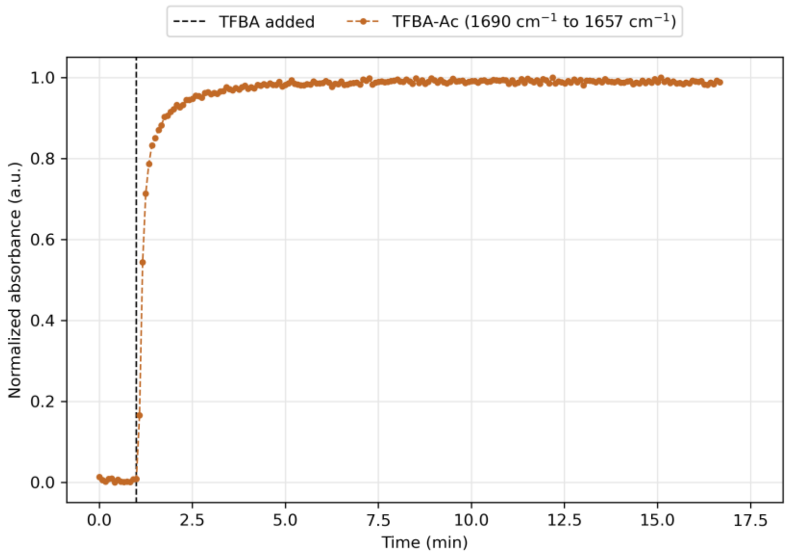

Figure S7. Normalized absorbance of TFBA-Ac over time during the acetylation reaction between TFBA and Np-Ac.

Batch experiment with addition of 24C8

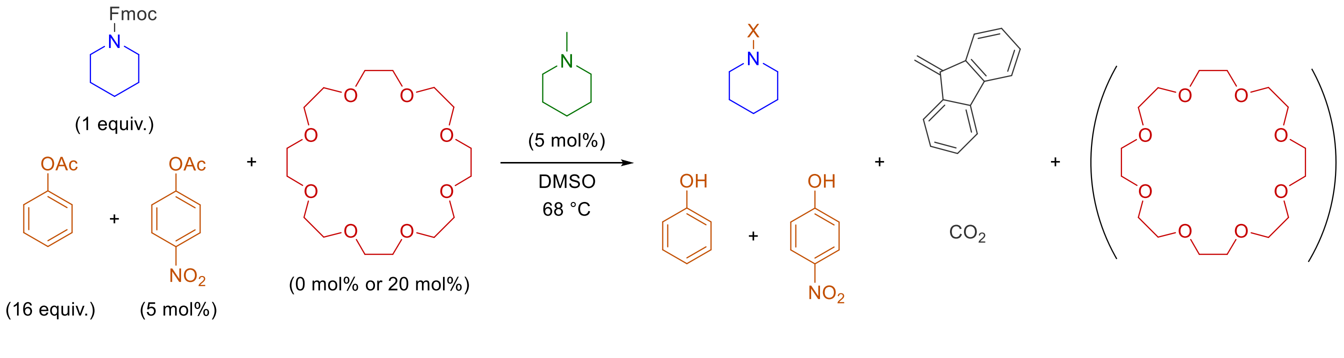

Scheme S12. Overall reaction for a piperidine pulse generation in batch initiated in the presence of 24C8.

Fmoc-pip (124 mg, 0.40 mmol, 1.0 equiv.), PhOAc (834 µL, 6.57 mmol, 16 equiv.), Np-Ac (3.68 mg, 0.02 mmol, 0.05 equiv.) and 24C8 (28.6 mg, 0.08 mmol, 0.20 equiv.) were added to a volumetric flask. The flask was filled with DMSO to a total volume of 4 mL. The solution was transferred to a three-necked flask in an oil bath and the temperature was adjusted to 68 °C and monitored using an external temperature probe. *N*-methylpiperidine (2.4 µL, 0.02 mmol, 0.05 equiv.) was added. The reaction was monitored using an *in situ* FTIR probe for 30 minutes, taking measurements every 30 seconds. The absorbance over time of Fmoc-pip (area: 1712 cm^{-1} to 1668 cm^{-1}, baseline: 1716 cm^{-1} to 1664 cm^{-1}), Pip-Ac (area: 1657 cm^{-1} to 1615 cm^{-1}, baseline: 1661 cm^{-1} to 1611 cm^{-1}) and DBF (area: 795 cm^{-1} to 775 cm^{-1}, baseline: 797 cm^{-1} to 773 cm^{-1}) was determined using the *two-point baseline* integration function in *iC IR 7.1* software (Figure 8).

Following the same steps, a solution was prepared with Fmoc-pip (124 mg, 0.40 mmol, 1.0 equiv.), PhOAc (816 µL, 6.4 mmol, 16 equiv.) and Np-Ac (3.68 mg, 0.02 mmol, 0.05 equiv.) in a volumetric flask and filled with DMSO to a total volume of 4 mL. The reaction was initiated by addition of *N*-methylpiperidine (2.4 µL, 0.02 mmol, 0.05 equiv.) and followed by FTIR.

No significant changes between the reaction with and without 24C8 addition were observed (Figure S9).

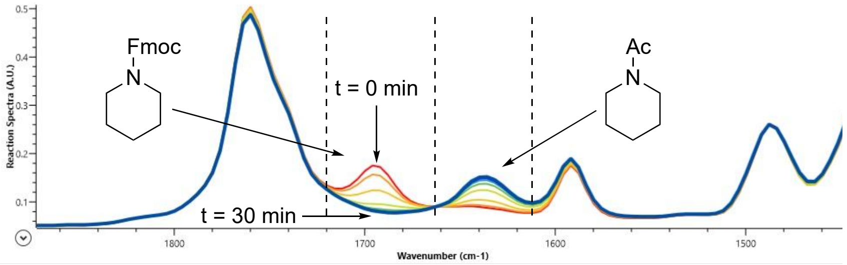

Figure S8. Zoom-in of example FTIR spectra obtained while monitoring a piperidine pulse in batch initiated in the presence of 24C8. Spectra range from 0 minutes (red) to 30 minutes (blue) of reaction time. The monitored bands corresponding to Pip-Fmoc and Pip-Ac are marked with dashed lines.

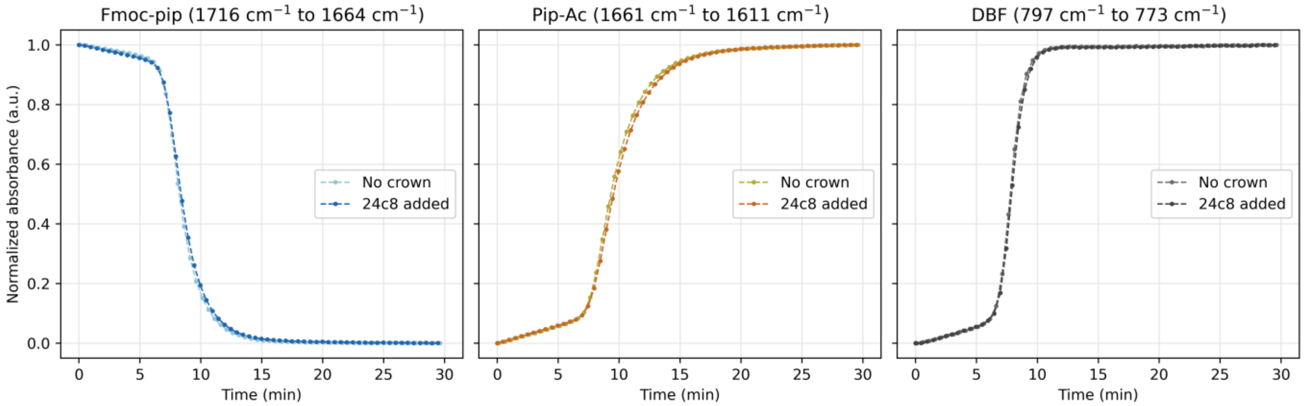

Figure S9. Normalized absorbance over time of Fmoc-pip (blue trace), Pip-Ac (yellow trace) and DBF (black trace) resulting from a batch experiment performed with and without addition of the 24C8 cargo.

9. Oscillatory cargo release

To achieve periodic behavior, the cargo release system needs to operate under flow conditions. For this purpose, we used sample collection for GC-FID analysis. The reaction was sampled from either the reactor directly or the outflow via a 6-way valve (Figure S10). In the former case, the outflow rate was adjusted to compensate for the volume removed by sampling.

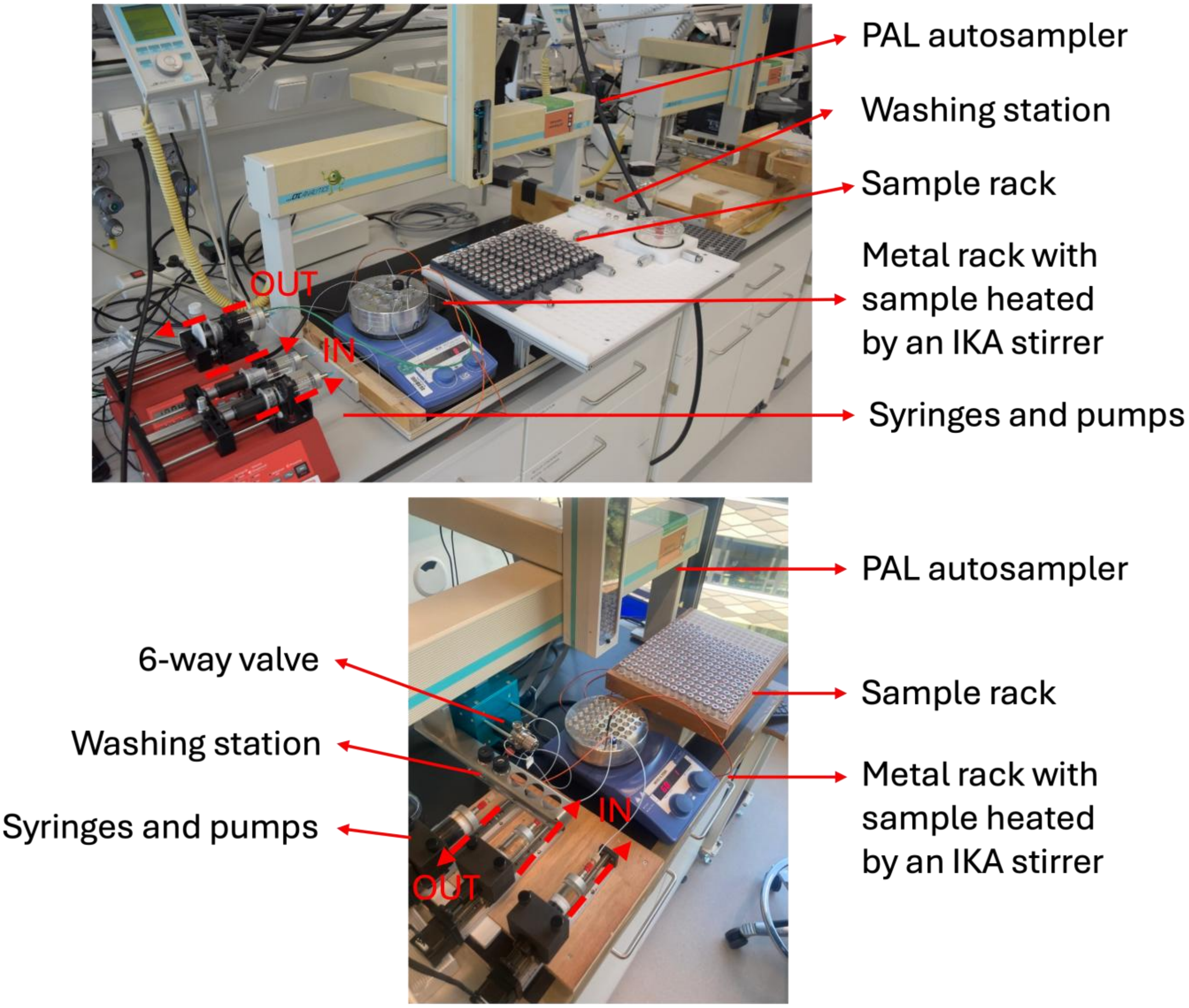

Figure S10. Annotated pictures of the experimental set-ups used for flow experiments. Top: configuration for sampling from the reactor. Bottom: configuration for sampling from the 6-way valve.

The concentration of Pip-Prop (corresponding to free piperidine), Pip-Ac, TFBA-Prop and TFBA-Ac was determined from the GC-FID chromatograms against the TMB internal standard using previously prepared calibration curves (Figure S11). For carriers **1** and **3**, the concentration of the cargo could not be determined directly as the rotaxane carriers decompose in the GC-FID instrument releasing additional cargo. Thus, the cargo concentration was calculated as the sum of TFBA-Prop and TFBA-Ac concentrations (Figure S12). For carrier **2**, the cargo concentration was monitored directly. Finally, in all plots, the moment of filling the reactor to the desired reaction volume was treated as the start of the reaction (t = 0 min).

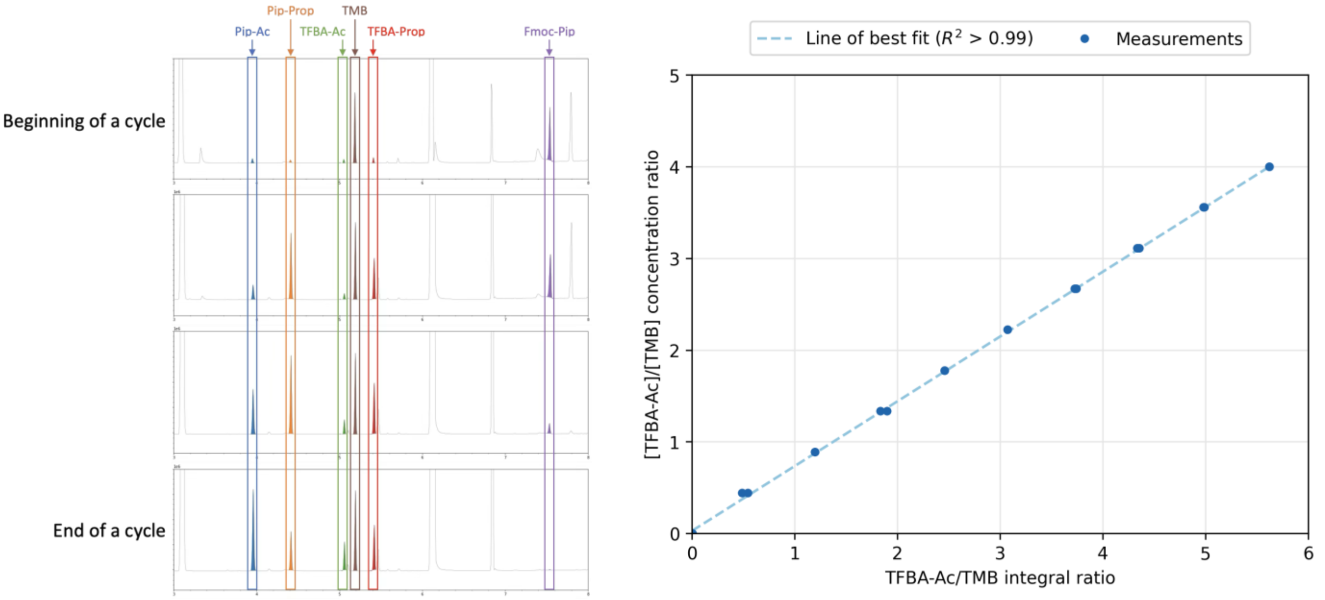

Figure S11. Left: Example chromatograms obtained from GC-FID measurements during an oscillatory cycle. Peaks and their integration are color-coded according to the corresponding compound. Right: Example calibration curve for determining compound concentration (here: TFBA-Ac) in GC-FID.

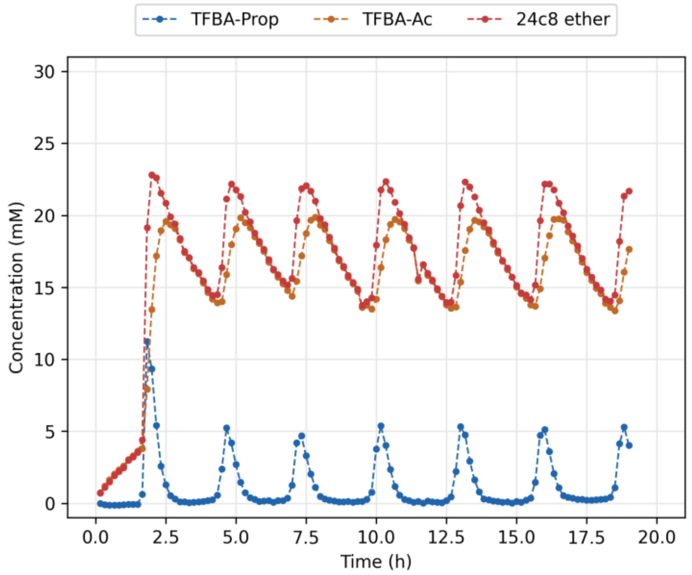

Figure S12. Concentration of TFBA-Prop, TFBA-Ac and 24C8 calculated as the sum of amides concentration in a flow experiment entry 2 in Table S5 (first 19 h).

General procedure for oscillatory cargo release monitored by GC-FID (D)

A quencher stock solution (0.1 M) was prepared by dissolving Np-Prop in EtOAc. GC vials for sample collection were filled with the quencher stock solution (0.8 mL each). A reagent stock solution was prepared in a volumetric flask by dissolving Fmoc-pip (1.0 equiv.), carrier (0.50 equiv.), PhOAc (18 equiv.), Np-Ac (0.30 equiv. to 0.40 equiv.) and TMB (0.50 equiv.) in DMSO. Equal volume of the trigger stock solution was prepared in a volumetric flask by dissolving *N*-methylpiperidine (4 mol% – 5 mol%) in DMSO. The solutions were transferred into separate syringes equipped with PTFE tubing and placed in syringe pumps. An extraction syringe with PTFE tubing was placed in a syringe pump. To prime the outflow, the line was placed in a vial with DMSO, and the extraction pump was turned on. A reactor vial was placed in a metal rack heated by an IKA stirrer, with temperature adjusted to 68 °C and monitored using an external temperature probe. The inflow lines were placed inside the reactor vial. While stirring, the vial was filled to the target volume by pumping the reagent and trigger solutions: 4 mL (each 12.0 mL/h for 10 minutes) – sampling reaction mixture, 1 mL (each 0.500 mL/min for 1 minute) – sampling outflow. The inflow rates were adjusted to reach the desired space velocity, the outflow line was placed in the vial, and reagent flow was started. Syringes and lines were kept above 20 °C with a space heater. The reaction was monitored by sampling the reaction mixture or the outflow using an autosampler. Samples were collected into GC vials filled with the quencher stock solution and analyzed by GC-FID.

Screening conditions for oscillatory cargo release

Table S5. Summary of tested conditions for oscillatory cargo release with carrier **1**[a]

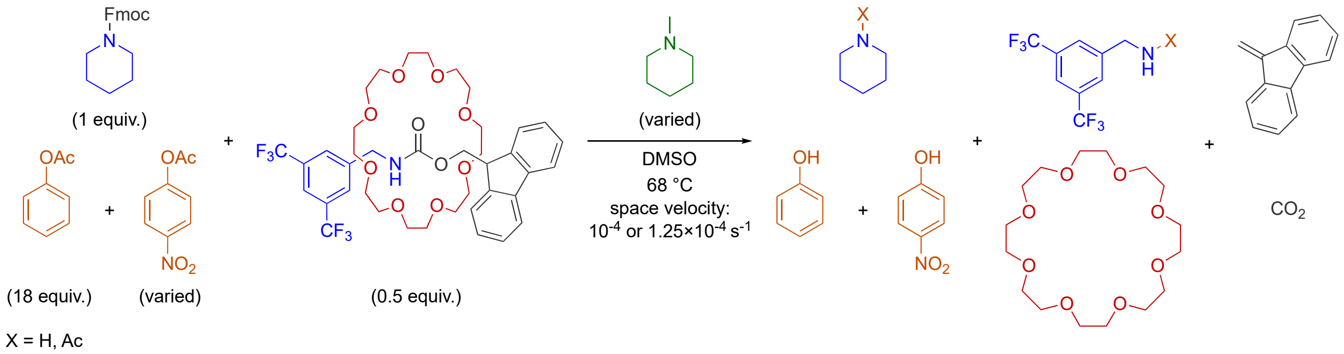

Entry	Np-Ac [equiv.]	*N*-pip [mol%]	v [s^{-1}]	Sustained oscillations?	Period [h]	Amplitude [mM]	Cargo release [%]	Sampling
**1**	0.30	5.0	10^{-4}	No	1.6	NA	NA	Reactor
**2**[b]	0.35	4.5	10^{-4}	Yes	2.8	8.1	45	Outflow
**3**	0.40	4.5	10^{-4}	Yes	4.0	11	45	Outflow
**4**	0.30	4.5	10^{-4}	No	1.7	NA	NA	Reactor
**5**	0.30	4.5	1.25×10^{-4}	Yes	1.5	3.3	44	Outflow
**6**	0.30	4.0	1.25×10^{-4}	No	1.5	NA	NA	Outflow

[a]Reaction conditions: 0.1 M Fmoc-pip, carrier **1** (0.5 eq.), trigger (4.0-5.0 mol%), fast inhibitor (0.30-0.40 equiv.), slow inhibitor (18 equiv.) in DMSO at 68 °C with space velocity: v = 10^{-4} s^{-1} or 1.25×10^{-4} s^{-1}. Monitored by sampling, quenching with 4-nitrophenyl propionate, and analysis via GC-FID with an internal standard. [b]Results calculated based only on the first 19 h.

While it may seem from Table S5 that the lack of sustained oscillations is caused by sampling from the reactor, we also conducted other reactions not presented in this work, both for this and other oscillating systems. There we could achieve sustained oscillations when sampling from the reactor. Furthermore, with this method, only 8 µL samples are taken every 10-15 minutes, while the reactor volume is 4 mL making it highly unlikely that the sampling interferes with the reaction.

Results for conditions shown in entry 1, 2 (first 19 h), 3 and 5 are presented in Figure 6b-f. All measurements obtained for conditions entry 2 are shown in Figure S13. Data corresponding to entry 4 is plotted in Figure S14. For entry 6, due to GC-FID column malfunction, the integration of TFBA-Ac signal was disrupted resulting in inaccurate calculation of the 24C8 ether concentration (Figure S15). A plot summarizing the tested conditions is shown in Figure S16.

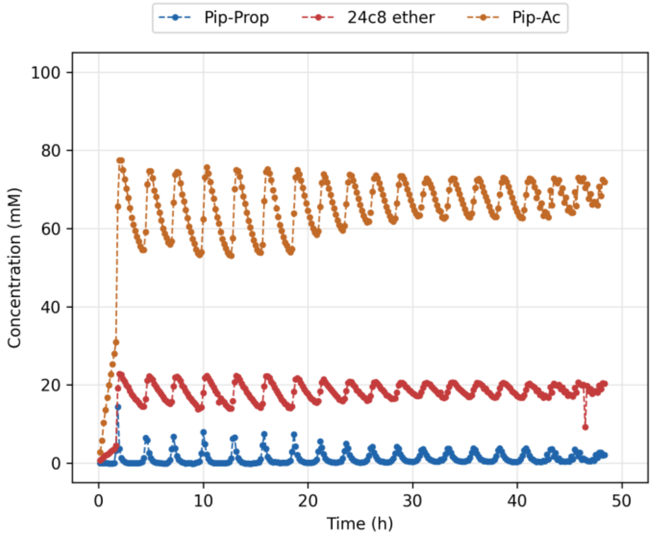

Figure S13. Results of a flow experiment performed under condition entry 2 (Table S3). After 19 h, due to temperature variation or syringe pump and in/out-flow line fatigue, the system switches to a new limit cycle.

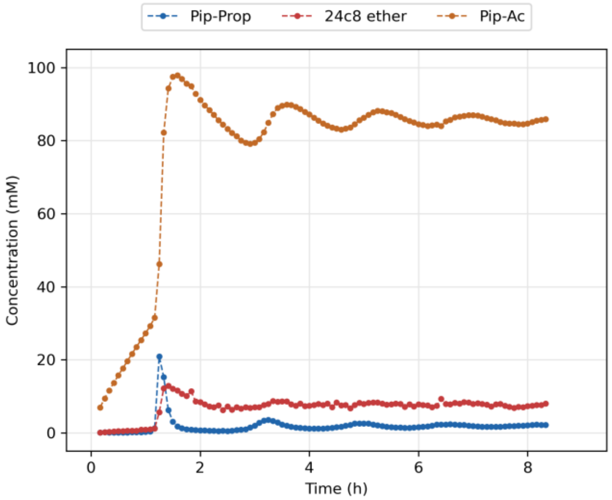

Figure S14. Results of a flow experiment performed under condition entry 4 (Table S5).

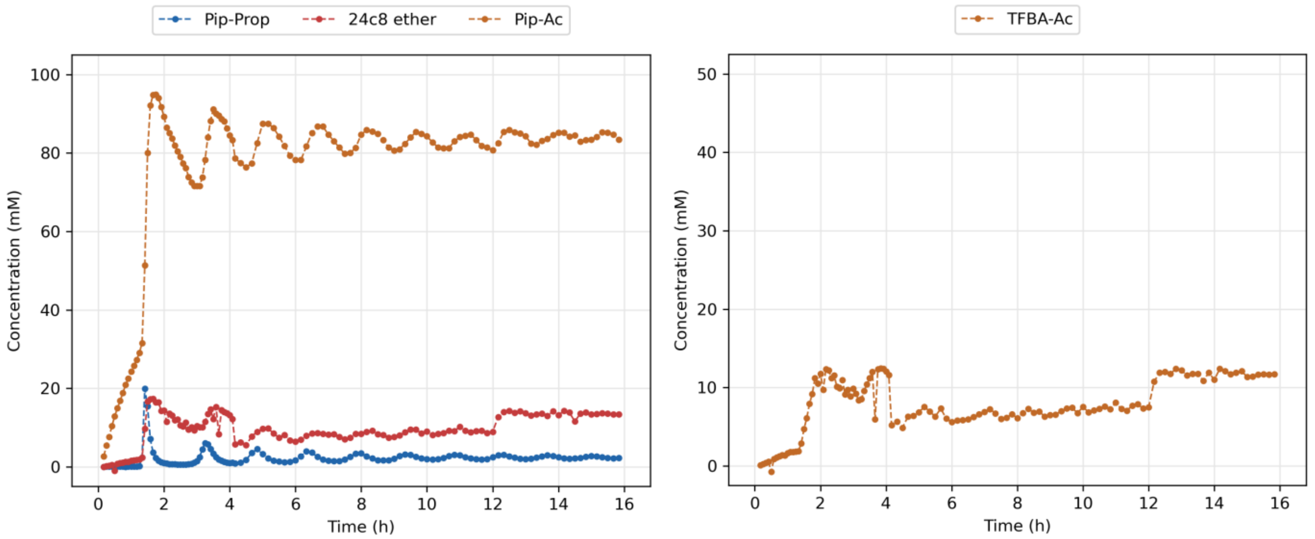

Figure S15. Results of a flow experiment performed under condition entry 6 (Table S5). Left: concentration of Pip-Prop, 24C8 and Pip-Ac over time. Right: concentration of TFBA-Ac as detected by GC-FID with faulty integration.

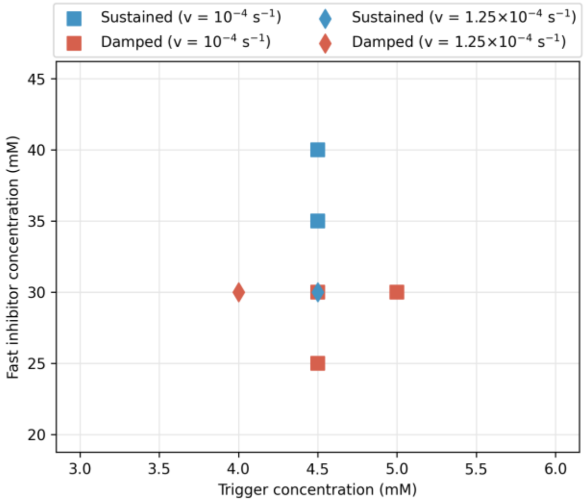

Figure S16. Summary of tested conditions. Each point corresponds to a fast inhibitor and trigger concentration combination. Square points indicate v = 10^{-4} s^{-1} and diamonds v = 1.25×10^{-4} s^{-1}. Blue color corresponds to sustained oscillations and red to damped oscillations.

Screening conditions for oscillatory cargo release

Table S6. Summary of tested conditions for oscillatory cargo release with carriers **2** and **3**[a]

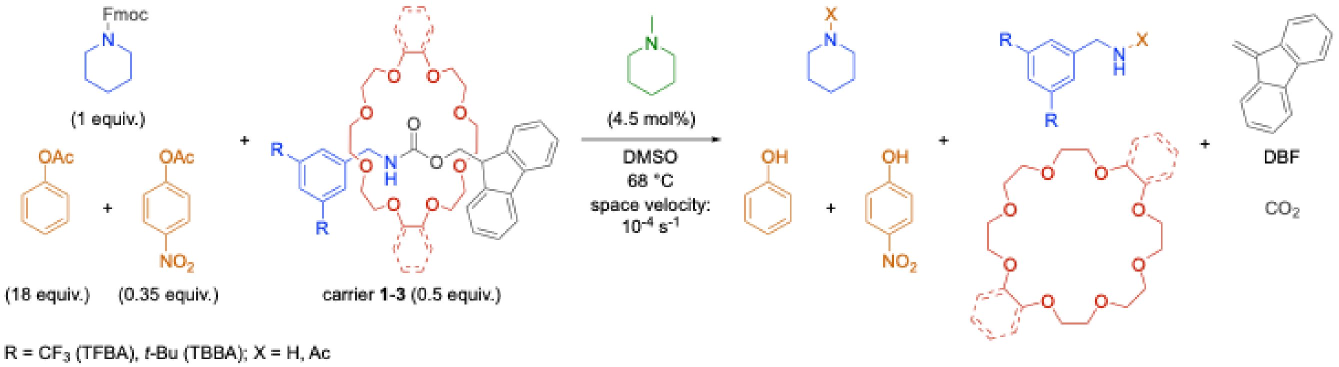

Carrier	Sustained oscillations?	Period [h]	Amplitude [mM]	Cargo release [%]	Sampling
**2**	Yes	2.5	8.5	56	Outflow
**3**	Yes	3.6	7.3	29	Outflow

[a]Reaction conditions: 0.1 M Fmoc-pip, carrier **2**-**3** (0.5 eq.), trigger (4.5 mol%), fast inhibitor (0.35 equiv.), slow inhibitor (18 equiv.) in DMSO at 68 °C with space velocity: v = 10^{-4} s^{-1}. Monitored by sampling, quenching with 4-nitrophenyl propionate, and analysis via GC-FID with an internal standard.

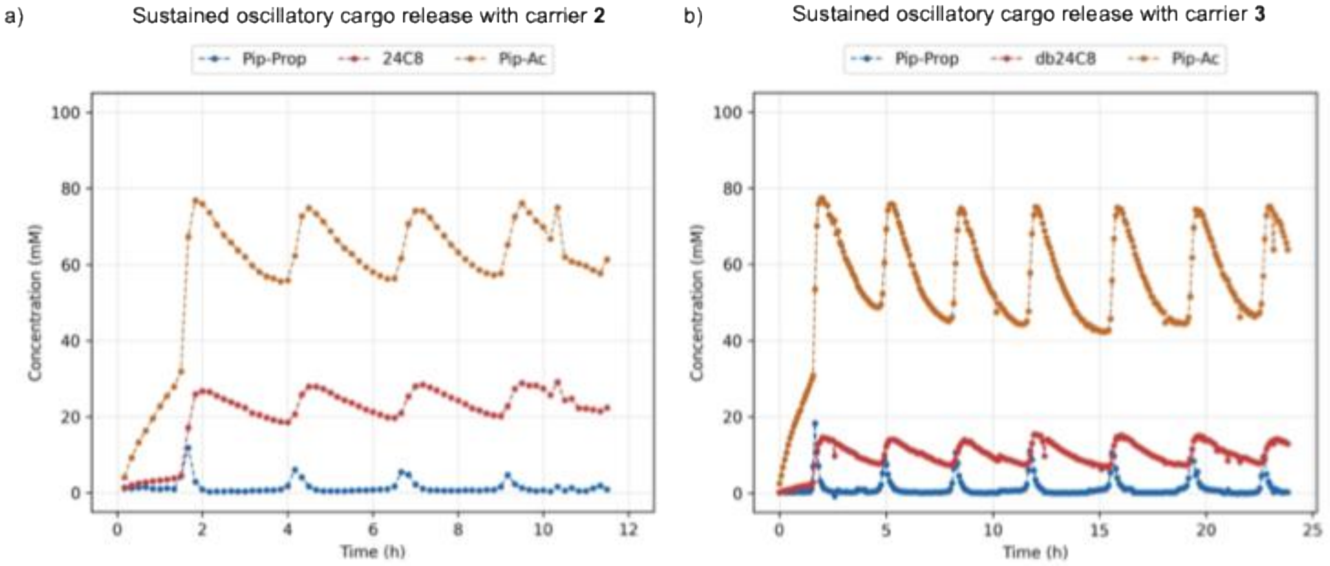

Figure S17. Results of flow experiements with carriers 2 and 3. (a) Sustained oscillations for carrier 2 and conditions listed in Table S6. (b) Sustained oscillations for carrier 3 and conditions listed in Table S6.

10. NMR spectra of isolated compounds

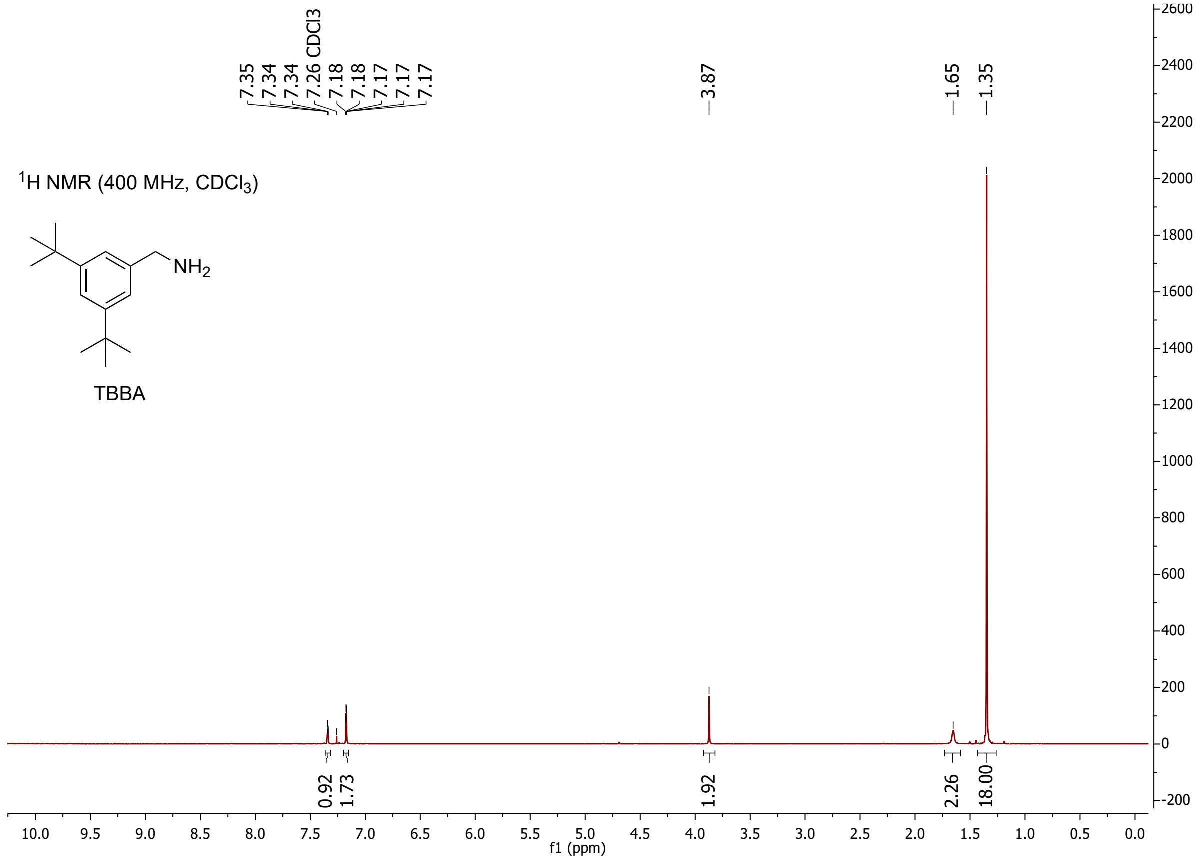

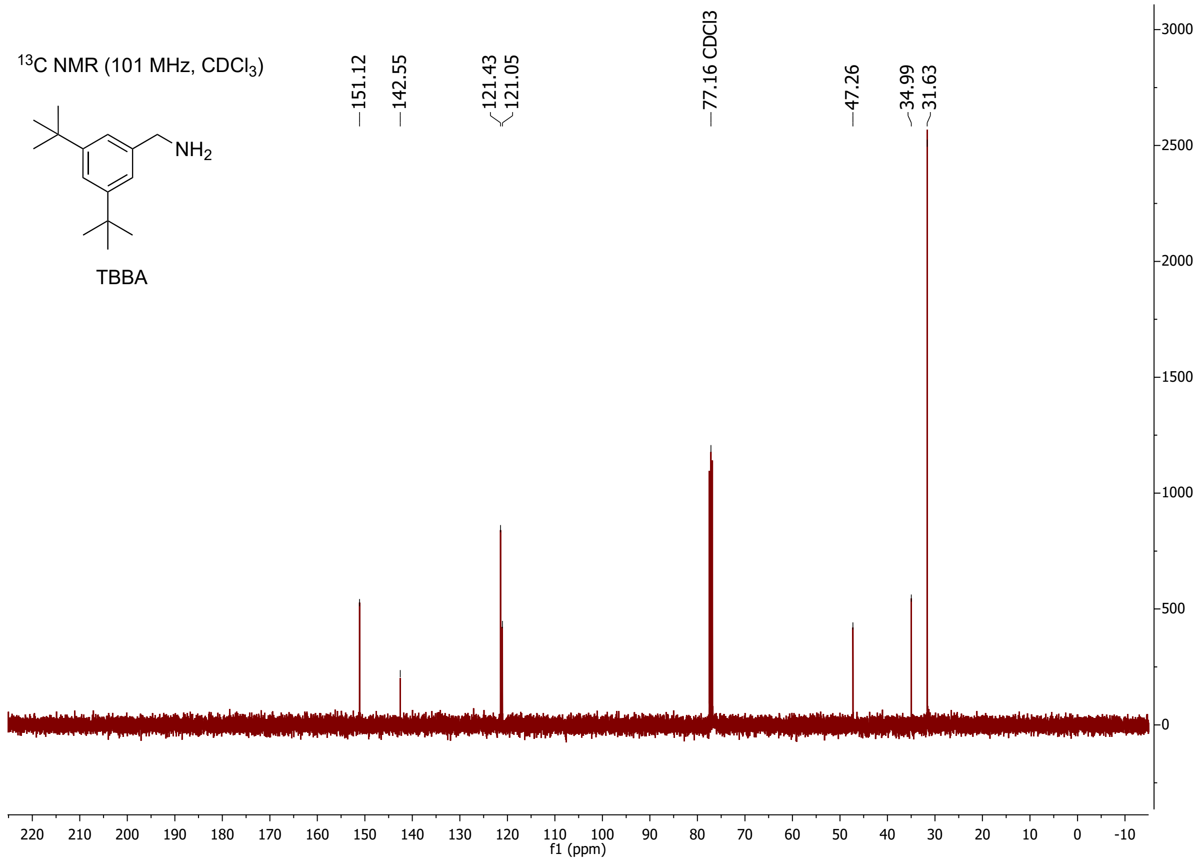

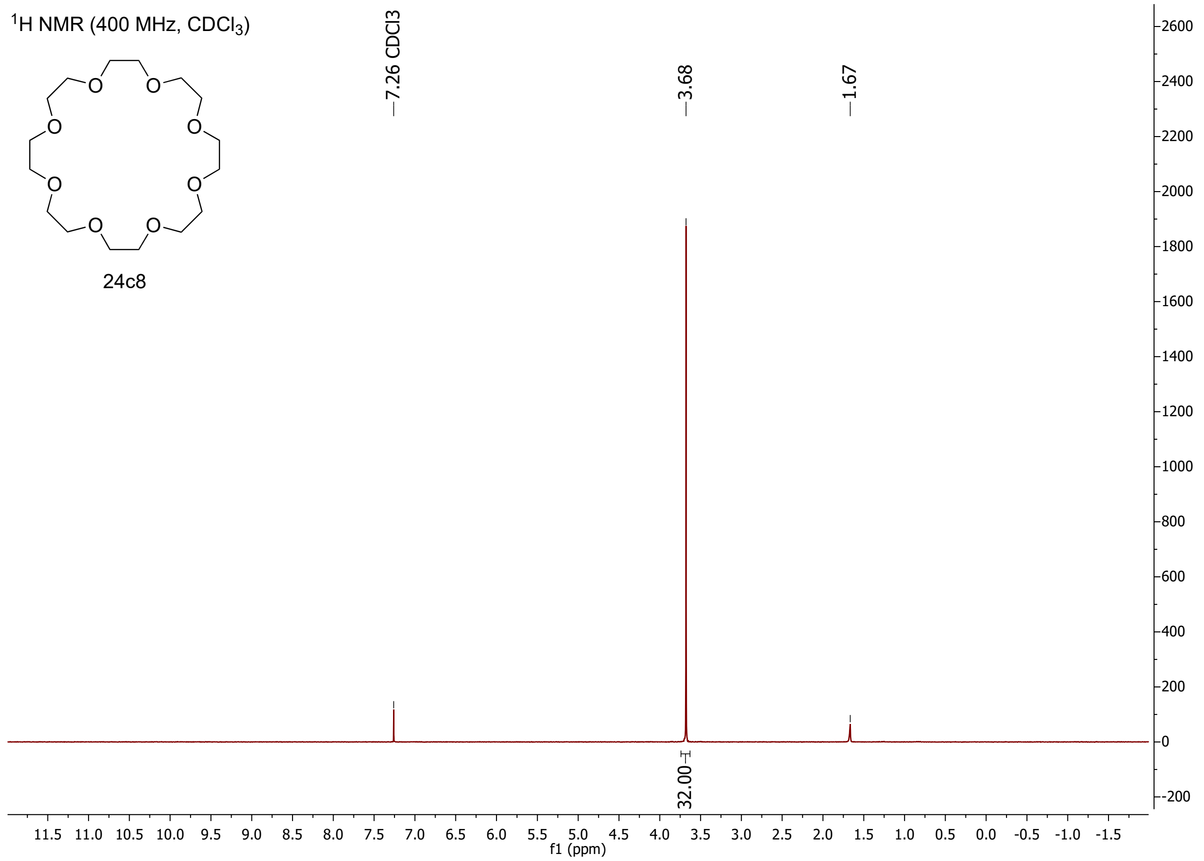

^{1}H NMR (400 MHz, $CDCl_3$)
24c8
7.26 CDCl3
3.68
1.67
32.00
2600
2400
2200
2000
1800
1600
1400
1200
1000
800
600
400
200
0
-200
11.5 11.0 10.5 10.0 9.5 9.0 8.5 8.0 7.5 7.0 6.5 6.0 5.5 5.0 4.5 4.0 3.5 3.0 2.5 2.0 1.5 1.0 0.5 0.0 -0.5 -1.0 -1.5
f1 (ppm)

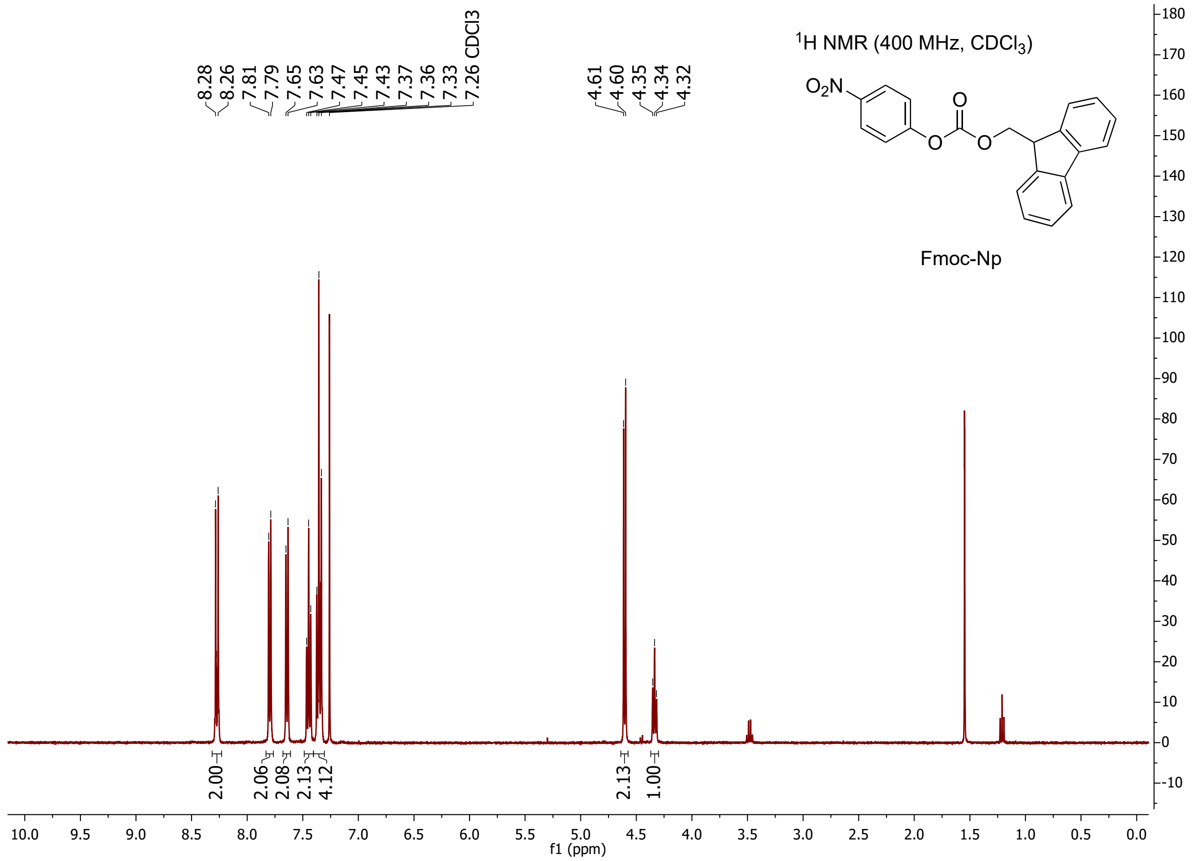

8.28
8.26
7.81
7.79
7.65
7.63
7.47
7.45
7.43
7.37
7.36
7.33
7.26 CDCl3
4.61
4.60
4.35
4.34
4.32
^1H NMR (400 MHz, $CDCl_3$)
O_2N
O
O
O
Fmoc-Np
2.00
2.06
2.08
2.13
4.12
2.13
1.00
f1 (ppm)

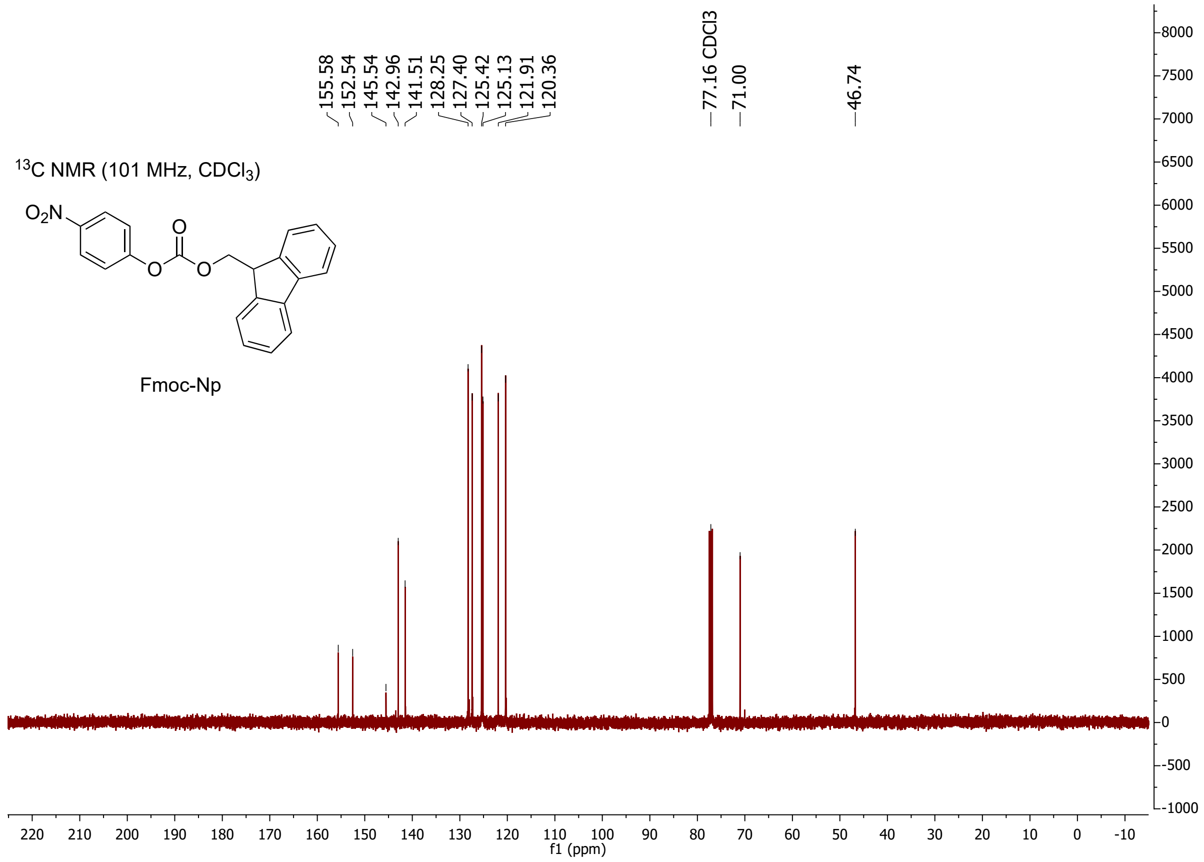

155.58
152.54
145.54
142.96
141.51
128.25
127.40
125.42
125.13
121.91
120.36
77.16 CDCl3
71.00
46.74
^{13}C NMR (101 MHz, $CDCl_3$)
O_2N
O
O
O
Fmoc-Np
f1 (ppm)

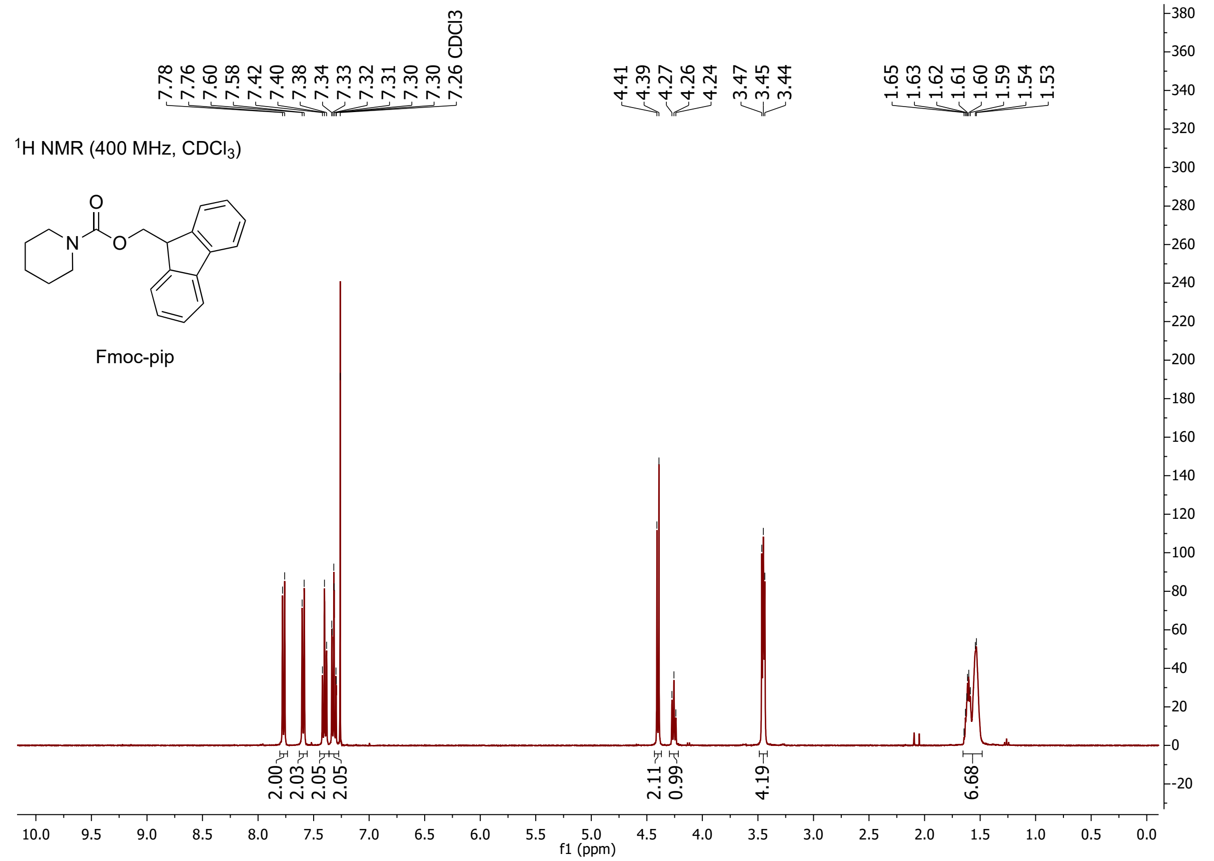
7.78
7.76
7.60
7.58
7.42
7.40
7.38
7.34
7.33
7.32
7.31
7.30
7.30
7.26 CDCl3
4.41
4.39
4.27
4.26
4.24
3.47
3.45
3.44
1.65
1.63
1.62
1.61
1.60
1.59
1.54
1.53
1H NMR (400 MHz, CDCl3)
O
N
O
Fmoc-pip
2.00
2.03
2.05
2.05
2.11
0.99
4.19
6.68
f1 (ppm)

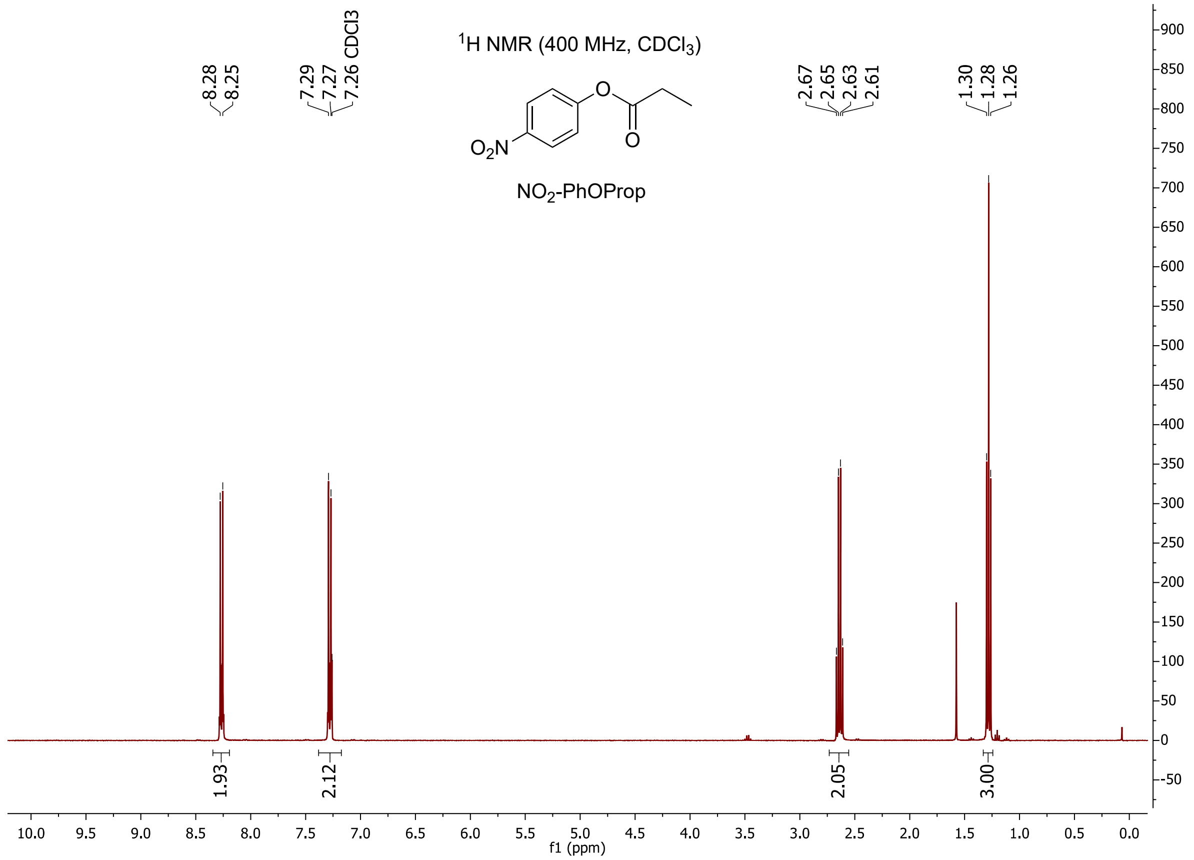

^1H NMR (400 MHz, $CDCl_3$)
NO_2-PhOProp
8.28
8.25
7.29
7.27
7.26 CDCl3
2.67
2.65
2.63
2.61
1.30
1.28
1.26
1.93
2.12
2.05
3.00
f1 (ppm)

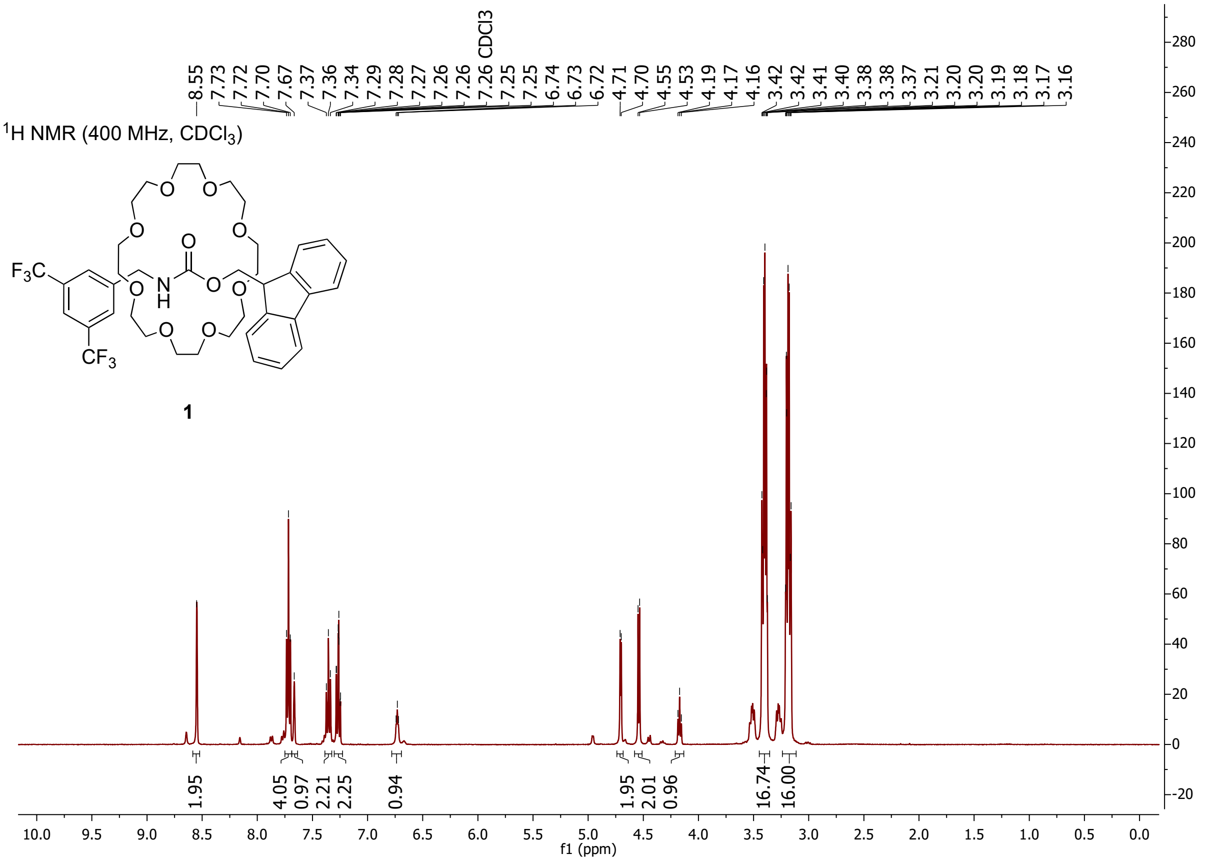

8.55 7.73 7.72 7.70 7.67 7.37 7.36 7.34 7.29 7.28 7.27 7.26 7.26 7.26 CDCl3 7.25 7.25 6.74 6.73 6.72 4.71 4.70 4.55 4.53 4.19 4.17 4.16 3.42 3.42 3.41 3.40 3.38 3.38 3.37 3.21 3.20 3.20 3.19 3.18 3.17 3.16
^{1}H NMR (400 MHz, $CDCl_3$)
F_3C
CF_3
O
N
H
1
1.95 4.05 0.97 2.21 2.25 0.94 1.95 2.01 0.96 16.74 16.00
f1 (ppm)

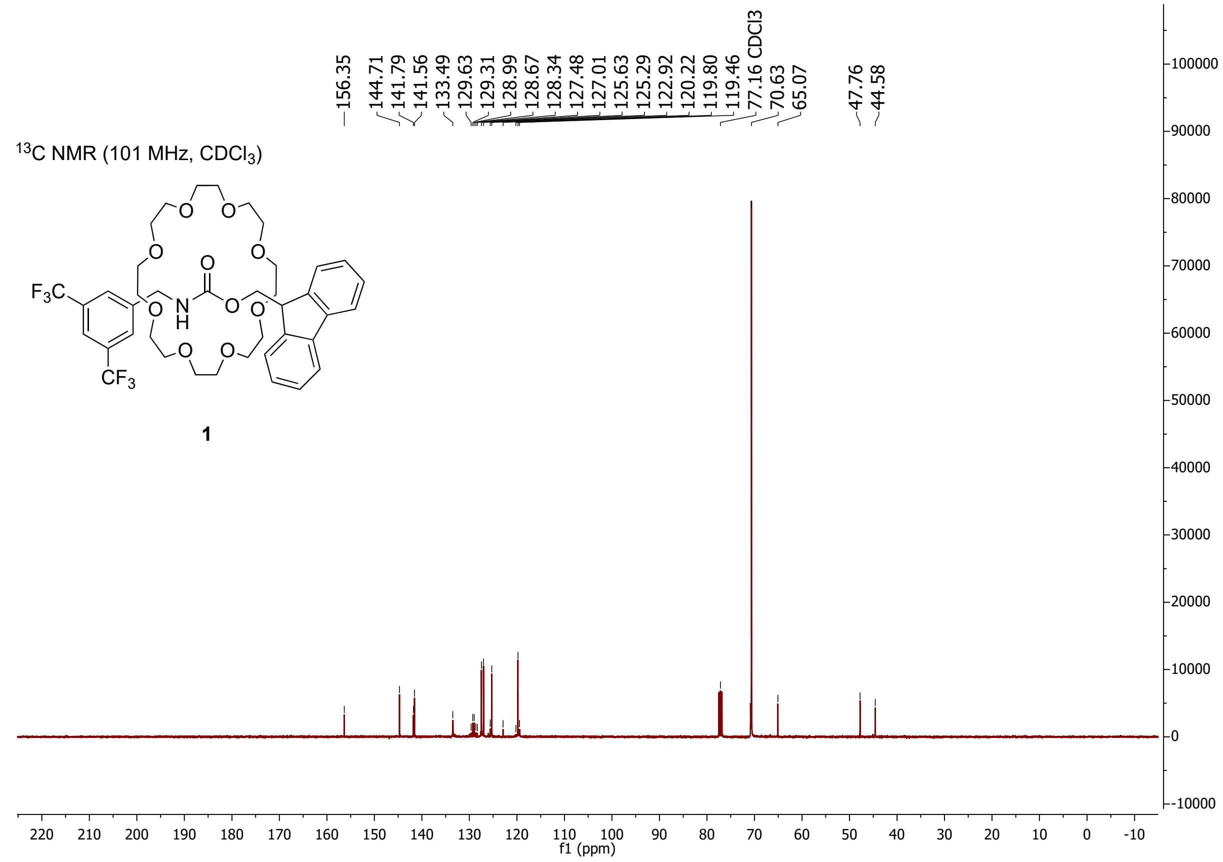

156.35 144.71 141.79 141.56 133.49 129.63 129.31 128.99 128.67 128.34 127.48 127.01 125.63 125.29 122.92 120.22 119.80 119.46 77.16 CDCl3 70.63 65.07 47.76 44.58
^{13}C NMR (101 MHz, $CDCl_3$)
F_3C
CF_3
O
N
H
1
f1 (ppm)

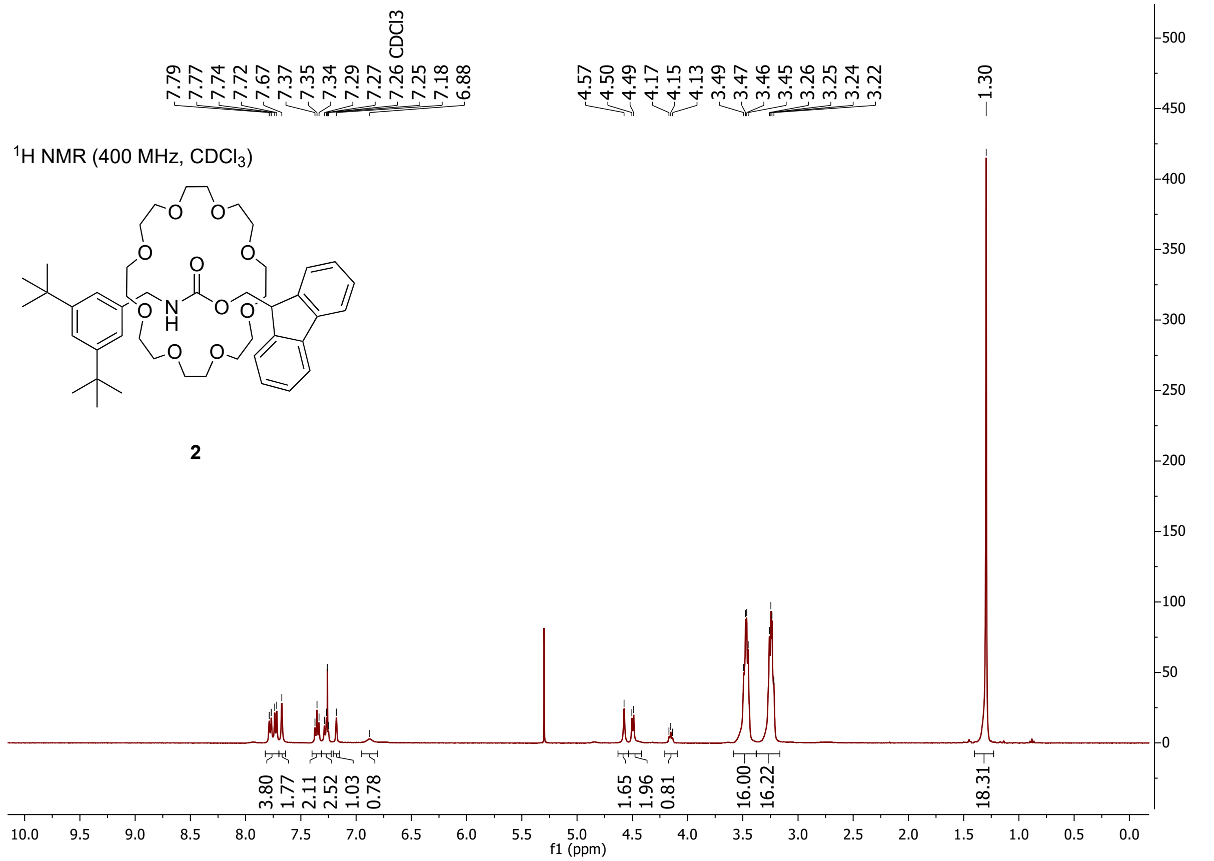
1H NMR (400 MHz, CDCl3)
2
7.79 7.77 7.74 7.72 7.67 7.37 7.35 7.34 7.29 7.27 7.26 CDCl3 7.25 7.18 6.88
4.57 4.50 4.49 4.17 4.15 4.13 3.49 3.47 3.46 3.45 3.26 3.25 3.24 3.22
1.30
3.80 1.77 2.11 2.52 1.03 0.78 1.65 1.96 0.81 16.00 16.22 18.31
f1 (ppm)

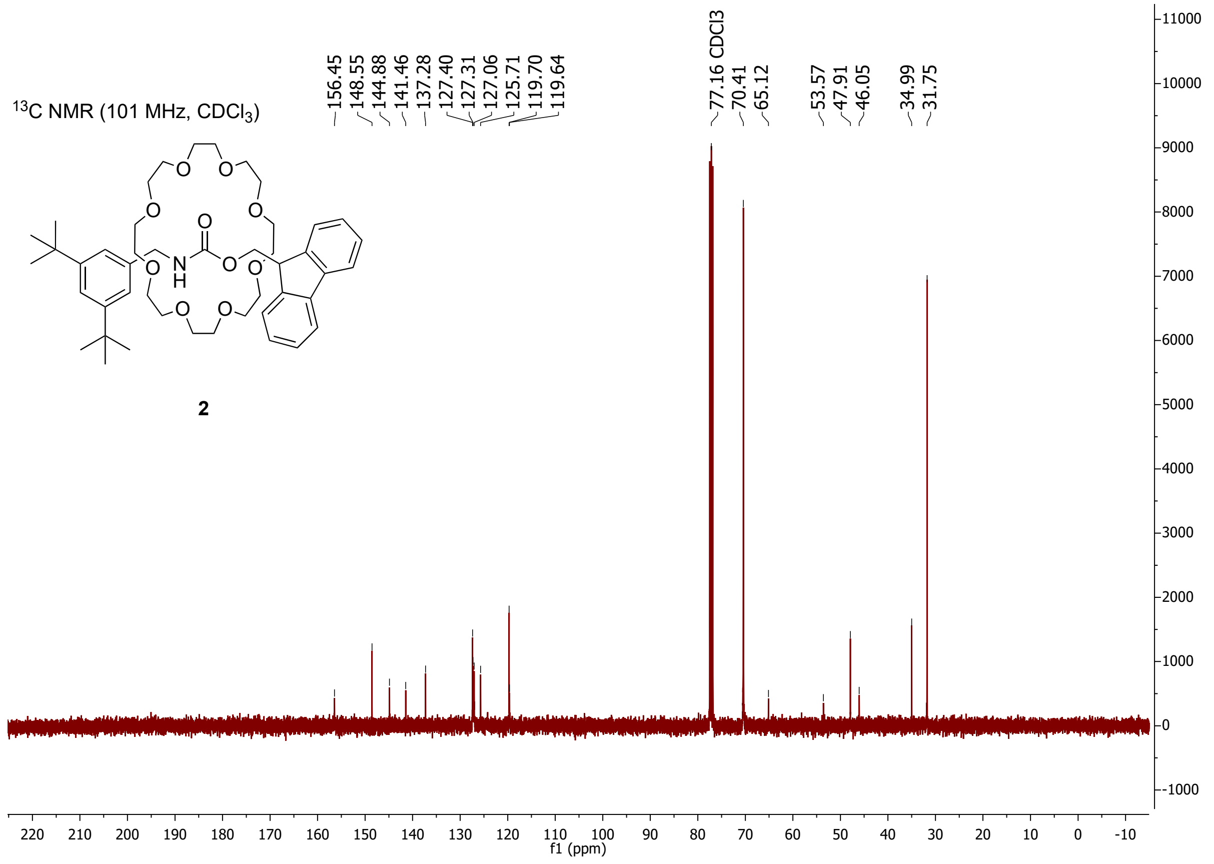
13C NMR (101 MHz, CDCl3)
2
156.45 148.55 144.88 141.46 137.28 127.40 127.31 127.06 125.71 119.70 119.64
77.16 CDCl3 70.41 65.12
53.57 47.91 46.05
34.99 31.75
f1 (ppm)

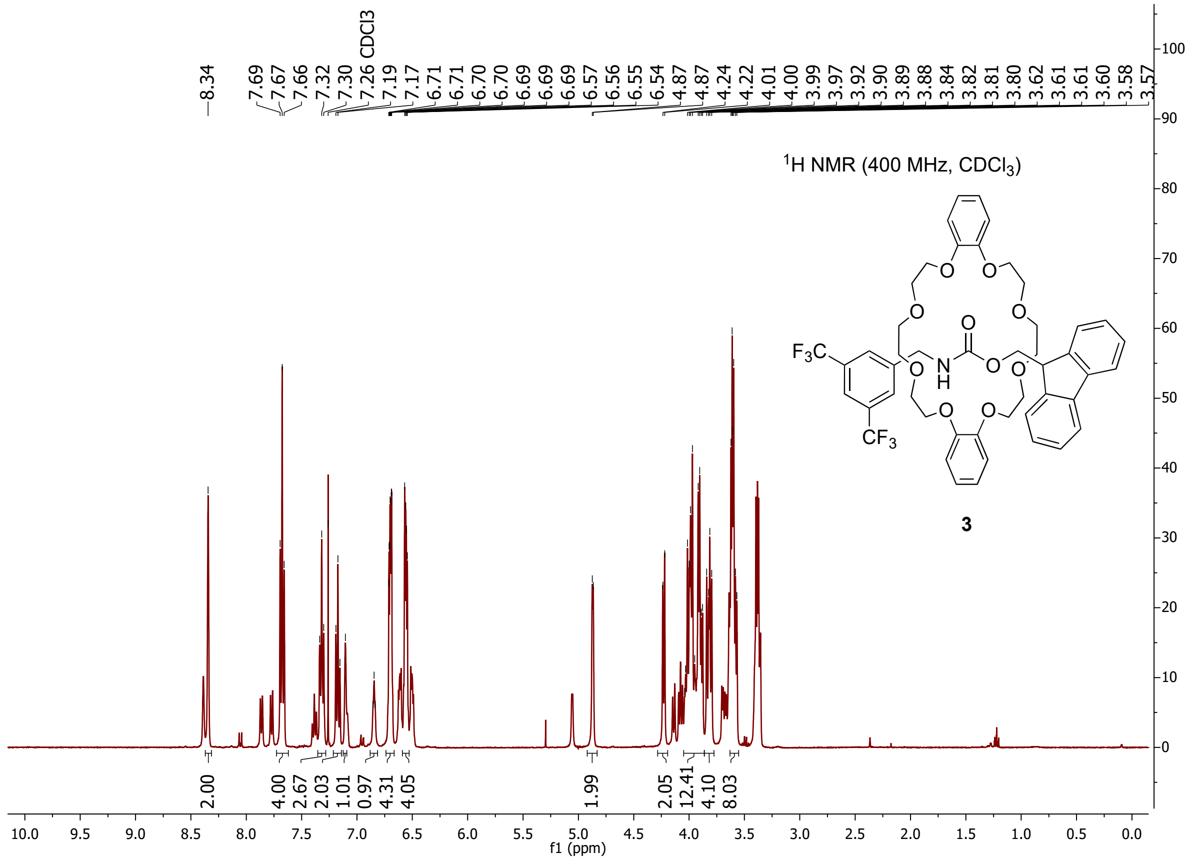

8.34
7.69
7.67
7.66
7.32
7.30
7.26 CDCl3
7.19
7.17
6.71
6.71
6.70
6.69
6.69
6.69
6.57
6.56
6.55
6.54
4.87
4.87
4.24
4.22
4.01
4.00
3.99
3.97
3.92
3.90
3.89
3.88
3.84
3.82
3.81
3.80
3.62
3.61
3.61
3.60
3.58
3.57
^{1}H NMR (400 MHz, CDCl$_3$)
F_3C
CF_3
3
2.00
4.00
2.67
2.03
1.01
0.97
4.31
4.05
1.99
2.05
12.41
4.10
8.03
f1 (ppm)

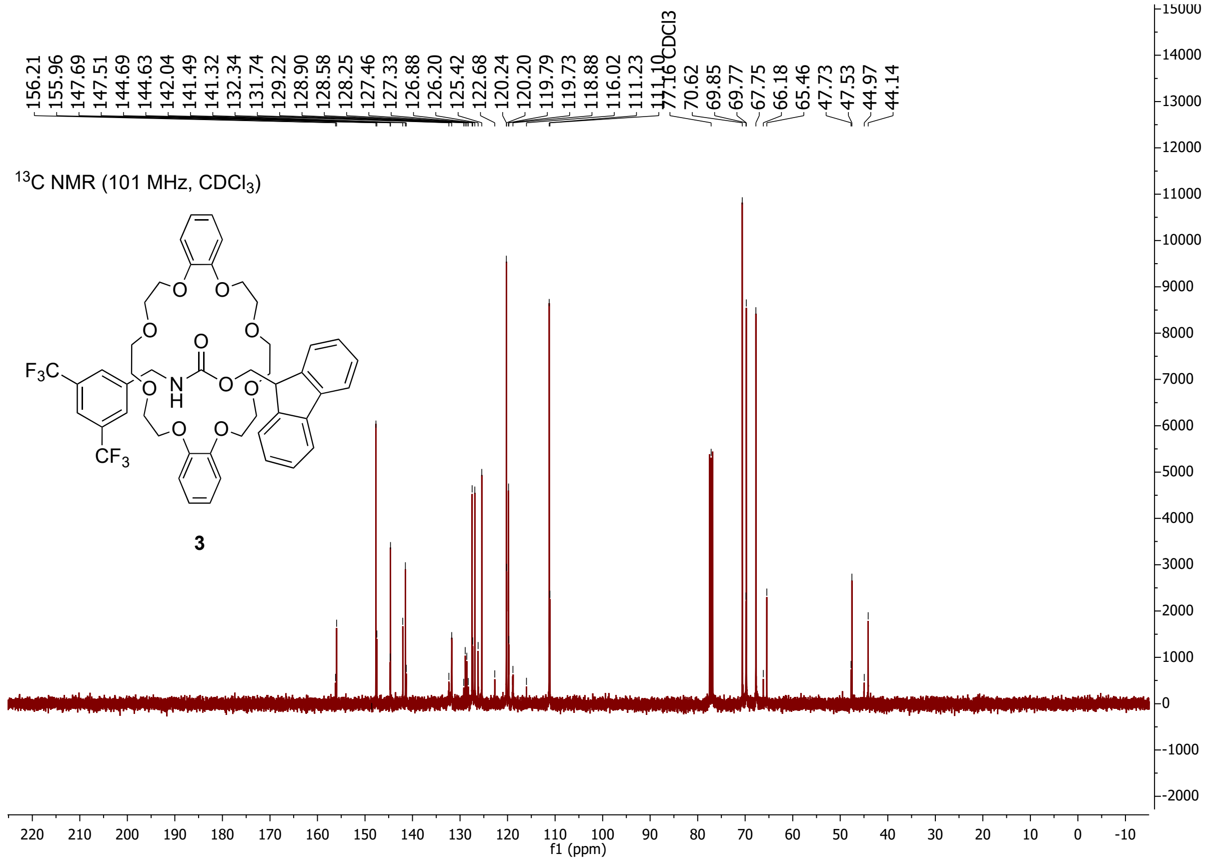

156.21
155.96
147.69
147.51
144.69
144.63
142.04
141.49
141.32
132.34
131.74
129.22
128.90
128.58
128.25
127.46
127.33
126.88
126.20
125.42
122.68
120.24
120.20
119.79
119.73
118.88
116.02
111.23
111.10
77.16 CDCl3
70.62
69.85
69.77
67.75
66.18
65.46
47.73
47.53
44.97
44.14
^{13}C NMR (101 MHz, CDCl$_3$)
F_3C
CF_3
3
f1 (ppm)

10. References

(1) Kootstra, J.; Sneep, J. L.; Le Cacheux, M. L. C.; Petryczkiewicz, K. D.; Harutyunyan, S. R. *Development of an affordable automation platform for organic chemistry labs.* Manuscript in preparation.

(2) Grunder, S.; Torres, D. M.; Marquardt, C.; Błaszczyk, A.; Krupke, R.; Mayor, M. Synthesis and Optical Properties of Molecular Rods Comprising a Central Core-Substituted Naphthalenediimide Chromophore for Carbon Nanotube Junctions. *Eur. J. Org. Chem.* **2010**, *2011* (3), 478–496. DOI:10.1002/ejoc.201001415.

(3) Talanov, V. S.; Bartsch, R. A. Improved Preparation of 24-Crown-8. *Synth. Commun.* **1999**, *29* (20), 3555–3560. DOI: 10.1080/00397919908085989.

(4) Merrifield, R. B.; Bach, A. E. 9-(2-Sulfo)Fluorenylmethyloxycarbonyl Chloride, a New Reagent for the Purification of Synthetic Peptides. *J. Org. Chem.* **1978**, *43* (25), 4808–4816. DOI: 10.1021/jo00419a021.

(5) ter Harmsel, M.; Maguire, O. R.; Runikhina, S. A.; Wong, A. S. Y.; Huck, W. T. S.; Harutyunyan, S. R. A Catalytically Active Oscillator Made from Small Organic Molecules. *Nature* **2023**, *621* (7977), 87–93. DOI: 10.1038/s41586-023-06310-2.

(6) Ghosh, U.; Ganessunker, D.; Sattigeri, V. J.; Carlson, K. E.; Mortensen, D. J.; Katzenellenbogen, B. S.; Katzenellenbogen, J. A. Estrogenic Diazenes: Heterocyclic Non-Steroidal Estrogens of Unusual Structure with Selectivity for Estrogen Receptor Subtypes. *Bioorg. Med. Chem.* **2003**, *11* (4), 629–657. DOI: 10.1016/s0968-0896(02)00309-7.

(7) Fielden, S. D. P.; Leigh, D. A.; McTernan, C. T.; Pérez- Saavedra, B.; Vitorica-Yrezabal, I. J. Spontaneous Assembly of Rotaxanes from a Primary Amine, Crown Ether and Electrophile. *J. Am. Chem. Soc.* **2018**, *140* (19), 6049–6052. DOI: 10.1021/jacs.8b03394.
